# Concerted Actions of a Thermo-labile Regulator and a Unique Intergenic RNA Thermosensor Control *Yersinia* Virulence

**DOI:** 10.1371/journal.ppat.1002518

**Published:** 2012-02-16

**Authors:** Katja Böhme, Rebekka Steinmann, Jens Kortmann, Stephanie Seekircher, Ann Kathrin Heroven, Evelin Berger, Fabio Pisano, Tanja Thiermann, Hans Wolf-Watz, Franz Narberhaus, Petra Dersch

**Affiliations:** 1 Institut für Mikrobiologie, Technische Universität Braunschweig, Braunschweig, Germany; 2 Abteilung Molekulare Infektionsbiologie, Helmholtz-Zentrum für Infektionsforschung, Braunschweig, Germany; 3 Department of Molecular Biology, Laboratory of Molecular Infectious Medicine, Umeå University, Umeå, Sweden; 4 Lehrstuhl für Biologie der Mikroorganismen, Ruhr-Universität Bochum, Bochum, Germany; Tufts University School of Medicine, United States of America

## Abstract

Expression of all *Yersinia* pathogenicity factors encoded on the virulence plasmid, including the *yop* effector and the *ysc* type III secretion genes, is controlled by the transcriptional activator LcrF in response to temperature. Here, we show that a protein- and RNA-dependent hierarchy of thermosensors induce LcrF synthesis at body temperature. Thermally regulated transcription of *lcrF* is modest and mediated by the thermo-sensitive modulator YmoA, which represses transcription from a single promoter located far upstream of the *yscW*-*lcrF* operon at moderate temperatures. The transcriptional response is complemented by a second layer of temperature-control induced by a unique *cis*-acting RNA element located within the intergenic region of the *yscW-lcrF* transcript. Structure probing demonstrated that this region forms a secondary structure composed of two stemloops at 25°C. The second hairpin sequesters the *lcrF* ribosomal binding site by a stretch of four uracils. Opening of this structure was favored at 37°C and permitted ribosome binding at host body temperature. Our study further provides experimental evidence for the biological relevance of an RNA thermometer in an animal model. Following oral infections in mice, we found that two different *Y. pseudotuberculosis* patient isolates expressing a stabilized thermometer variant were strongly reduced in their ability to disseminate into the Peyer's patches, liver and spleen and have fully lost their lethality. Intriguingly, *Yersinia* strains with a destabilized version of the thermosensor were attenuated or exhibited a similar, but not a higher mortality. This illustrates that the RNA thermometer is the decisive control element providing just the appropriate amounts of LcrF protein for optimal infection efficiency.

## Author Summary

Many important virulence genes remain silent at moderate temperatures in external environments and are rapidly and strongly induced by a sudden temperature upshift sensed upon host entry. Thermal activation of virulence gene transcription is frequently described, but post-transcriptional control mechanisms implicated in temperature-sensing and induction of virulence factor synthesis are less evident. Here, we present a novel two-layer regulatory system implicating a protein- and an RNA-dependent thermosensor controlling synthesis of the most crucial virulence activator LcrF (VirF) of pathogenic yersiniae. In this case, moderate function of a thermosensitive gene silencer is coupled with the more dominant action of a unique intergenic two-stemloop RNA thermometer. Thermally-induced conformational changes in this RNA element control the transition between a ‘closed’ and an ‘open’ structure which allows ribosome access and translation of the *lcrF/virF* transcript. This mechanism guarantees optimal virulence factor production during the course of an infection, ideal for survival and multiplication of yersiniae within their warm-blooded hosts. The hierarchical concept combining two temperature-sensing modules constitutes a new example of how bacterial pathogens use complementing strategies to allow rapid, energetically cheap and fine-tuned adaptation of their virulence traits.

## Introduction

Pathogenic yersiniae, including *Y. pestis,* the causative agent of the bubonic plague, and the two enteric species *Y. enterocolitica* and *Y. pseudotuberculosis* which cause gut-associated diseases (yersiniosis) such as enteritis, diarrhea and mesenterial lymphadenitis express different sets of virulence factors important for different stages of the infection process [1]–[2]. It is well known that most of the *Yersinia* virulence genes are tightly controlled in response to temperature [3].

Some of the early stage virulence factors, including the primary internalization factor invasin of both enteric *Yersinia* species, are mostly produced at moderate temperatures to allow efficient trespassing of the intestinal epithelial barrier shortly after infection [4]–[6]. These virulence genes are controlled by RovA, an intrinsic protein thermometer, which undergoes a conformation change upon a temperature shift from 25°C to 37°C, that reduces its DNA-binding capacity and renders it more susceptible to proteolysis [7]–[9].

Most other known *Yersinia* virulence genes remain silent outside the mammalian hosts and are only induced after host entry in response to the sudden increase in temperature. One important set of thermo-induced virulence factors is encoded on the 70 kb *Yersinia* virulence plasmid pYV (pCD1 in *Y. pestis*) [10]. These pathogenicity factors are crucial to avoid phagocytosis or other attacks by the innate immune defense system and comprise a type III secretion system (T3SS), the secreted *Yersinia* outer proteins (Yops) and regulatory components of the secretion system [11]–[13]. The Yop secretion genes (*ysc*) are organized in two operons *yscB-L* (*virC* operon) and *yscN-U,* or encoded elsewhere (e.g. *yscW, yscX, yscY* and *yscV*) on pYV [10] and are required for the formation of the T3S apparatus (injectisome). Body temperature (but not 20–25°C) and host cell contact trigger expression and translocation of Yop proteins by the T3S machinery into the cytoplasm of targeted host cells [14]–[17]. The Yop proteins can be divided into the group of translocators implicated in the formation of the translocation pore and the Yop effector proteins which manipulate numerous signal transduction pathways to prevent phagocytosis and the production of proinflammatory cytokines [18]–[21].

Expression of the majority of pYV-encoded virulence genes (*yadA*, *yop, lcr* and *ysc* genes for T3S and regulation) is induced by temperatures above 30°C in all pathogenic *Yersinia* species. Temperature-dependent induction of these genes requires the AraC-type DNA-binding protein LcrF (VirF in *Y. enterocolitica*) [22]–[24]. The LcrF protein contains a poorly conserved N-terminal oligomerization domain which is connected to a flexible highly conserved C-terminus with two helix-turn-helix DNA-binding motifs [25]. It exhibits high homology to the main regulator of T3S in *Pseudomonas aeruginosa*, ExsA and has been shown to bind specifically to TTTaGYcTtTat DNA motifs in the promoter regions of *yopE*, *lcrG*, *virC* and *yopH* [26]. The transcriptional activator LcrF is mainly produced at 37°C. Hoe and Goguen [27] showed that the *lcrF* mRNA produced in *E. coli* or *Y. pestis* could not be translated at 26°C, but was readily translated at 37°C. Based on predicted mRNA structure, these authors proposed that translation was dependent on melting of a stem-loop which sequestered the *lcrF* ribosomal binding site. Calculated thermal stability agreed well with observed translation, but no experimental work testing this hypothesis by manipulating stability of the structure was performed. In contrast, for *Y. enterocolitica* it has been reported that transcription of the *lcrF* homologous gene *virF* is increased at higher temperatures. This activation was shown to depend on topological changes and thermo-induced melting of intrinsically bent DNA identified upstream of the *lcrF/virF* gene [28]–[29]. Also chromosomally encoded factors that contribute to the temperature-dependent regulation of *yadA* and *yop* transcription have been identified in *Y. enterocolitica*. Below 30°C, induction of these virulence genes was only observed in the absence of the *Yersinia* modulator A (YmoA) [30]–[31]. YmoA belongs to the superfamily of nucleoid-associated proteins and shares 82% sequence identity with the regulator of “high hemolysin activity” (Hha) in *E. coli* and *Salmonella* [32]. The *E. coli* Hha protein represses the transcription of the *hlyCABD* operon encoding the pore-forming toxin hemolysin at moderate temperatures [33]–[34]. YmoA was shown to influence DNA supercoiling and forms heterodimers with the nucleoid-associated protein H-NS [33]–[36]. However, YmoA or H-NS binding to pYV promoter sequences has never been reported. Hence, the molecular mechanisms by which YmoA controls *yadA* and *yop* gene expression is still unclear. A recent analysis of type III secretion in *Y. pestis* indicated that regulated proteolysis of YmoA by the ATP-dependent Clp and Lon proteases plays an important role in the temperature-dependent expression of the type III secretion operons [37]. It was shown that YmoA is rapidly degraded at 37°C, but remains stable at environmental temperatures [37]. Whether the thermo-control mechanisms of LcrF vary between *Y. pestis* and *Y. enterocolitica* or whether they are connected, and if so, how they contribute to LcrF production in the different species remained elusive.

In this study, we investigated the molecular mechanism underlying thermoregulated production of the LcrF virulence regulator of *Y. pseudotuberculosis.* We found that concerted actions of the thermo-labile YmoA regulator protein and an unusual intergenic RNA thermosensor assured best possible production of LcrF for the highest infection efficiency. YmoA repressed *lcrF* transcription through sequences located within the 5′-UTR of *yscW* located upstream of the *lcrF* gene, and contributed moderately to the thermo-dependent production of LcrF. This activity is supplemented by a two-hairpin RNA thermometer composed of four uracil residues (fourU) that pair with the ribosome-binding site (AGGA) within the intergenic region of the *yscW-lcrF* mRNA. Using a mouse model system we provide evidence that this RNA thermosensor is mainly responsible for thermo-induced LcrF production, and show that its function is relevant for a high pathogenic potential, optimal survival and multiplication of *Yersinia* during infection.

## Results

### Temperature and YmoA-dependent regulation of *lcrF* transcription

**Figure 1:**
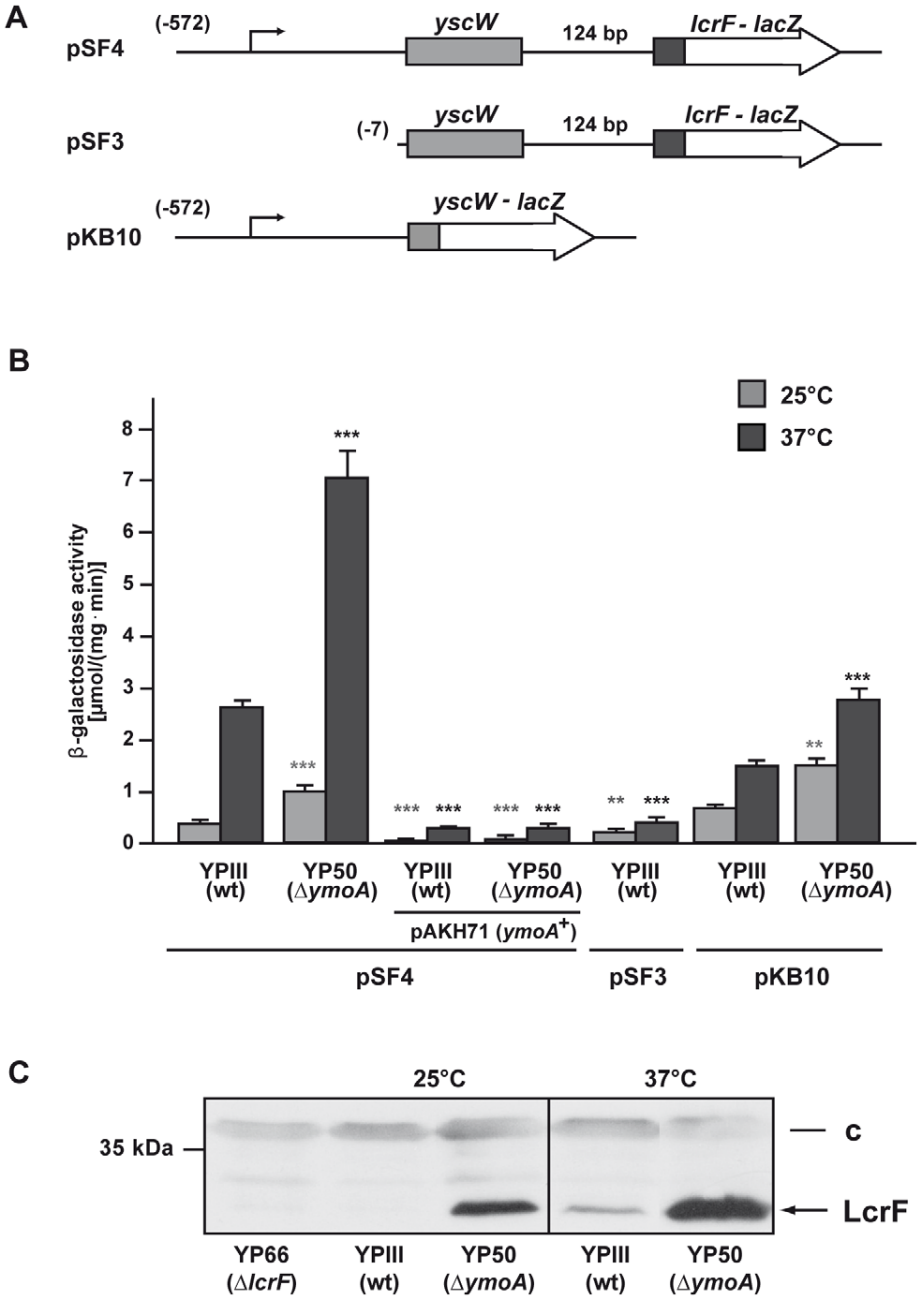
Expression of the *yscW-lcrF* operon in response to temperature. (**A**) Schematic presentation of the *yscW-lacZ* and *yscW-lcrF-lacZ* fusion plasmids. Numbers given in brackets represent the nucleotide positions of the 5′-end of the *yscW* regulatory region of the fusion constructs with respect to the start codon of *yscW*. The *yscW* gene is indicated in grey, the 5′-portion of the *lcrF* gene is given in black and the *lacZ* reporter gene is illustrated by a white arrow. (**B**) Strains YPIII and YP50 (Δ*ymoA*) harboring the *yscW-lacZ* (pKB10) or the *yscW-lcrF-lacZ* (pSF3 and pSF4) fusion plasmids ± pAKH71 (*ymoA* ^+^) were grown overnight in LB medium at 25°C or 37°C. β-Galactosidase activity from overnight cultures was determined and is given in µmol min^−1^ mg^−1^ for comparison. The data represent the average ± SD from at least three different experiments each done in duplicate. Data were analyzed by the Student's t test. Stars indicate the results that differed significantly from those of YPIII at the same temperature with ** (P<0.01), and *** (P<0.001). The activity of all reporter constructs differed significantly between 25°C and 37°C with P<0.001 (not shown). (**C**) Whole-cell extracts from overnight cultures of *Y. pseudotuberculosis* wild type and the mutant strains YP66 (Δ*lcrF*) and YP50 (Δ*ymoA*) grown at 25°C or 37°C were prepared and analysed by Western blotting with a polyclonal antibody directed against LcrF. A molecular weight marker is loaded on the left. A higher molecular weight protein (c) that reacted with the polyclonal antiserum was used as loading control.

The AraC-type transcriptional activator protein LcrF induces the expression of crucial *Yersinia* pathogenicity factors (e.g. YadA, T3SS and Yop effectors) in response to temperature. Initial efforts in this study to unravel the molecular control mechanisms of *lcrF* expression in *Y. pseudotuberculosis* demonstrated that the *lcrF* gene is organized in an operon with *yscW* (formerly named *virG*) located 124 bp upstream of the *lcrF* coding region on the *Yersinia* virulence plasmid pYV (Figure **1A**). As shown in Figure **1B**, a *yscW*-*lcrF-lacZ* (pSF4) and a *yscW-lacZ* (pKB10) translational fusion harboring the *yscW* regulatory region up to position −572 relative to the *yscW* start codon were expressed, whereas a construct carrying *yscW* sequences to position −7 (pSF3) was not. The *yscW*-*lcrF-lacZ* fusion was thermo-regulated in dependence of the YmoA protein. Expression was about 2-fold increased in the *ymoA* mutant strain and showed a significantly higher expression at 37°C than at 25°C (Figure **1B**). To confirm this result, western blot analysis was performed to detect the LcrF protein in cell extracts from the *Y. pseudotuberculosis* wildtype strain YPIII and the isogenic *ymoA* mutant YP50 grown at 25°C and 37°C. As shown in Figure **1C**, the LcrF protein could only be detected in extracts of the *ymoA* mutant but not in the wildtype strain when the bacteria were grown at 25°C. In contrast, LcrF production was significantly increased and detectable in both strains at 37°C, whereby the overall level of LcrF was significantly higher in the *ymoA*-deficient strain. This indicated that *lcrF* expression occurs from a temperature- and YmoA-dependent promoter located upstream of the *yscW* gene.

**Figure 2:**
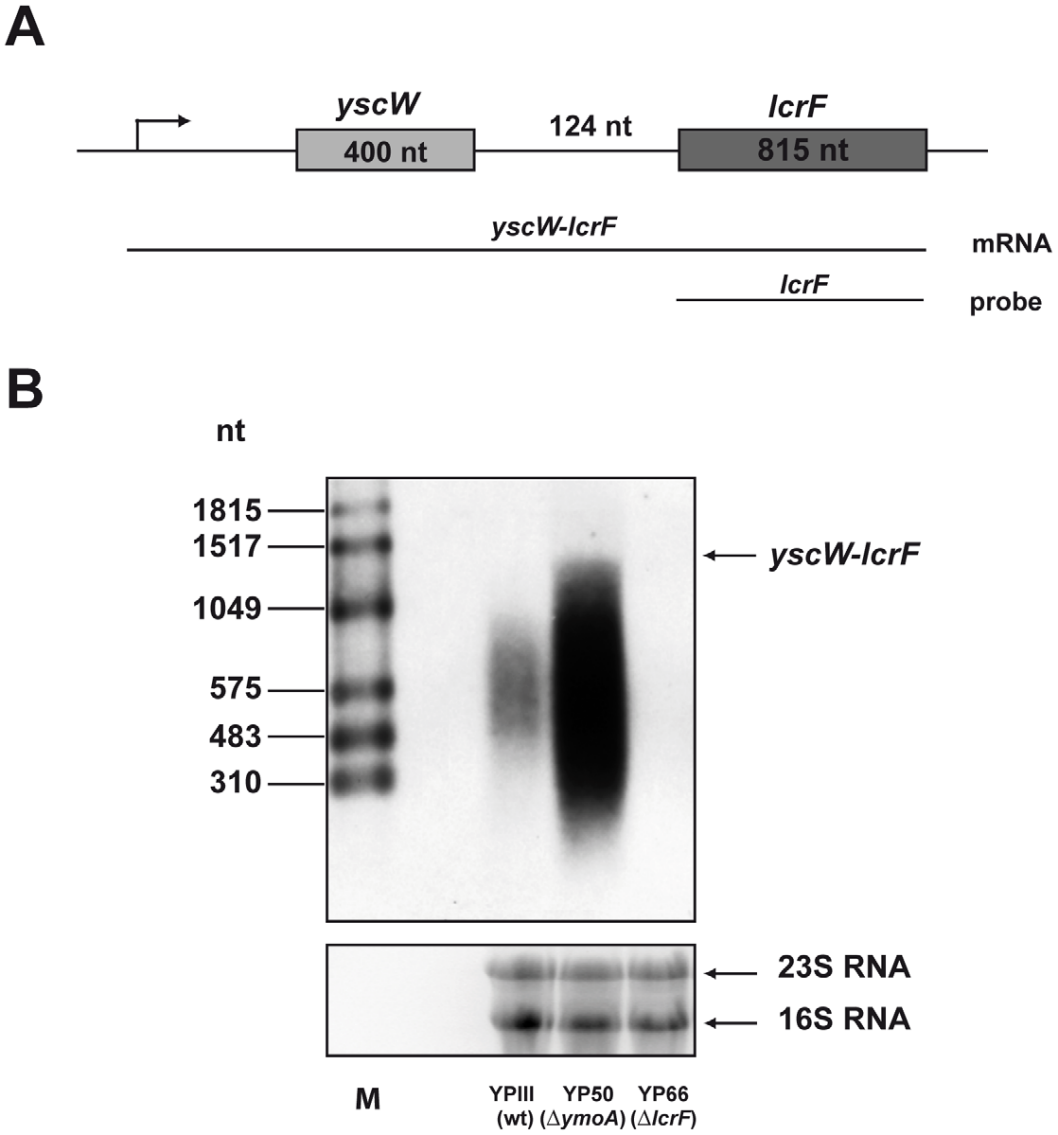
Analysis of the *yscW-lcrF* mRNA in wildtype and the *ymoA* mutant strain. (**A**) Schematic presentation of the *yscW-lcrF* operon, the *yscW-lcrF* mRNA and the *lcrF* probe used for Northern Blot analysis shown below. (**B**) Total RNA of YPIII, YP50 and YP66 was prepared, separated on a 1.2% agarose gel, transferred onto a Nylon membrane and probed with Digoxigenin-labelled PCR fragment encoding the *lcrF* gene. The 16S and 23S rRNAs are shown as RNA loading control. A RNA marker is loaded on the left.

In order to investigate *yscW-lcrF* transcription in more detail, total RNA of *Y. pseudotuberculosis* was prepared for Northern blot analysis using an *lcrF* specific probe (Figure **2**). The *yscW-lcrF* transcript was found to be highly unstable and was rapidly degraded into lower molecular weight transcripts in the wildtype (Figure **2**). In contrast, higher concentrations and higher molecular weight transcripts were detectable in the *ymoA* mutant strain consistent with the conclusion that *yscW* and *lcrF* originate from the same promoter. Moreover, as judged from the length of the *yscW-lcrF* transcript, the transcriptional start site appeared to be about 300 bp upstream of the *yscW* start codon, leading to the formation of a long 5′-untranslated region (5′-UTR).

**Figure 3:**
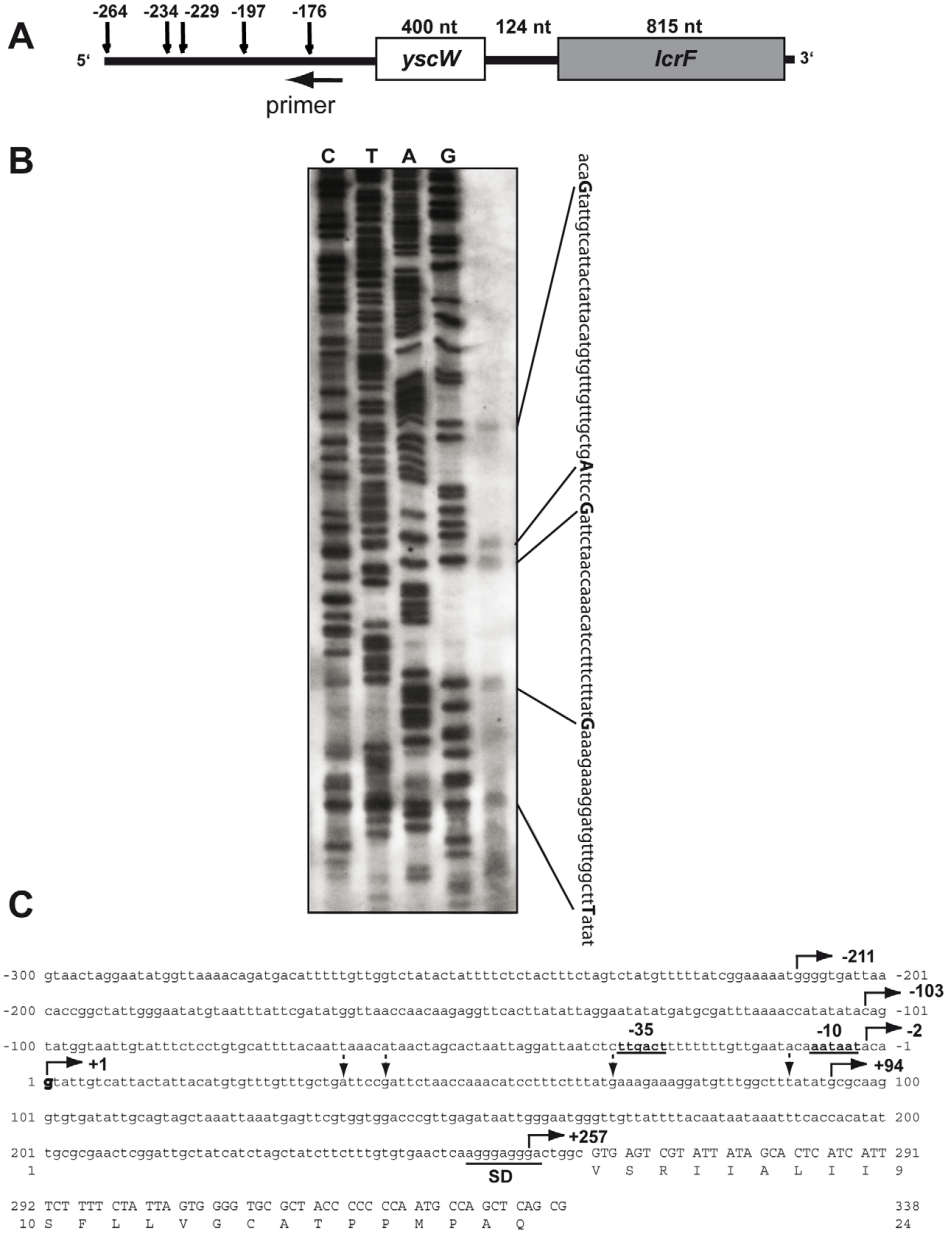
Mapping of the *lcrF* transcription start site by primer extension analysis. (**A**) The 5′-ends of the reverse transcription products are indicated by vertical arrows on the schematic presentation of the *yscW-lcrF* mRNA. The numbers indicate the position of the nucleotide of the 5′-ends with respect to the start codon of *yscW*. Location of the annealed primer used for primer extension is indicted by a horizontal arrow. (**B**) Total RNA of *Y. pseudotuberculosis* YPIII was prepared and used for primer extension analysis. Primers specific for the *yscW* coding sequence and 20 µg template RNA were applied for primer extension and obtained products were separated on a denaturing 6% polyacrylamide/urea gel. Sequencing reactions performed with the same primer are shown on the left. The sequence of the promoter region is shown on the right and identified 5′-end of the detected primer extension products are given in bold. (**C**) The regulatory region of the *yscW-lcrF* operon is shown. The broken arrows indicate the 5′-end points of the promoter deletion constructs and straight arrows show the 5′-end of the degradation products. The −35 and −10 region of the identified promoter is underlined, the transcriptional start site is given in bold, and the Shine-Dalgarno sequence (SD) is indicated.

To identify the *yscW-lcrF* promoter we performed primer extension analysis. We found that the transcription of the *yscW-lcrF* operon starts at a G found 264 nt upstream of the start codon GTG of *yscW* with a −35 and a −10 region of a typical σ^70^-dependent promoter (Figure **3**) leading to a 264 nt 5′-UTR. Several shorter reverse transcripts were consistent with the Northern results suggesting rapid processing of the *yscW-lcrF* transcript.

Increased expression of the *yscW-lacZ* and *yscW-lcrF-lacZ* fusions in the *ymoA* deficient *Yersinia* strain suggested that YmoA influences expression on the transcriptional level (Figure **1B** **, S1**). Continuous deletions of the promoter region showed that elimination of the identified promoter region by a 5′-upstream deletion up to position −2 abrogated transcription of the fusion construct and confirmed presence of a single promoter driving *yscW-lcrF* expression (Figure **S1A**, **3C**). Further analysis demonstrated that YmoA-dependency was maintained when sequences upstream of the *yscW* promoter were deleted, but it was lost when the 5′-UTR of *yscW* was removed (Figure **S1A,B**). This indicated that YmoA acts through sequences located downstream of the *yscW* promoter.

Next, we tested whether YmoA influence on *yscW*-*lcrF* was direct or involves (an)other regulatory factor(s). Experimental evidence support the hypothesis that members of the YmoA(Hha) protein family modulate gene expression by interacting with the nucleoid-structuring DNA-binding protein H-NS or its paralogs [36], [38]. However, other studies reported that the Hha/YmoA protein binds specifically to regulatory sequences of virulence genes. Unfortunately copurification of H-NS was not ruled out in these studies [39]–[41]. In order to test YmoA binding, YmoA was overexpressed and purified from *E. coli* strain KB4 (Δ*hns*, Δ*stpA*, Δ*hha*) deficient of all *E. coli* full-length and partial H-NS family proteins and used for band shift analysis with an *yscW* promoter fragment harboring the entire 5′-UTR. However, even at very high protein concentrations YmoA was not able to interact specifically with the *yscW* regulatory region (Figure **S2A**). In addition, we purified YmoA overexpressed in *E. coli* strain KB4 also expressing the *Y. pseudotuberculos*is *hns* gene. This YmoA protein sample included copurified H-NS*_Y.pstb_* (data not shown) and was able to interact specifically with the 5′-UTR sequences of the *yscW* gene (Figure **S2C**). We further expressed and purified H-NS*_Y.pstb_* in the absence of YmoA and found that also H-NS*_Y.pstb_* alone is capable to interact with the *yscW* regulatory sequences (Figure **S2B**). This indicated that YmoA influences *yscW*-*lcrF* expression directly and this involves heterocomplex formation with H-NS.

To confirm these data we also analyzed whether YmoA influence on thermal regulation of LcrF is lost, when the 5′-UTR important for H-NS/YmoA binding is absent. To do so, we compared expression of the *yscW-lcrF-lacZ* construct and a derived deletion variant of this fusion (*yscW*(Δ13–241)-*lcrF-lacZ*) at 25°C and 37°C. We found that the expression level is still thermoregulated, but *lcrF* transcription became independent of YmoA (Figure **S2D**). This clearly demonstrated that YmoA influences expression of *lcrF* via the 5′-UTR region of the *yscW* gene.

### Post-transcriptional control of *lcrF* thermoregulation

The YmoA protein of *Y. pestis* was shown to be subject to proteolysis by the Lon- and ClpP proteases at 37°C but not at 25°C [37], and this post-translational control was also observed for YmoA in *Y. pseudotuberculosis* YPIII (K. Böhme, unpublished results). However, expression of the *yscW-lcrF-lacZ* fusion and LcrF synthesis was still thermoregulated in the *ymoA*-deficient strain (Figure **1B,C**), suggesting that contribution of YmoA to *lcrF* thermoregulation is rather small and predominantly mediated by an additional YmoA-independent control mechanism.

**Figure 4:**
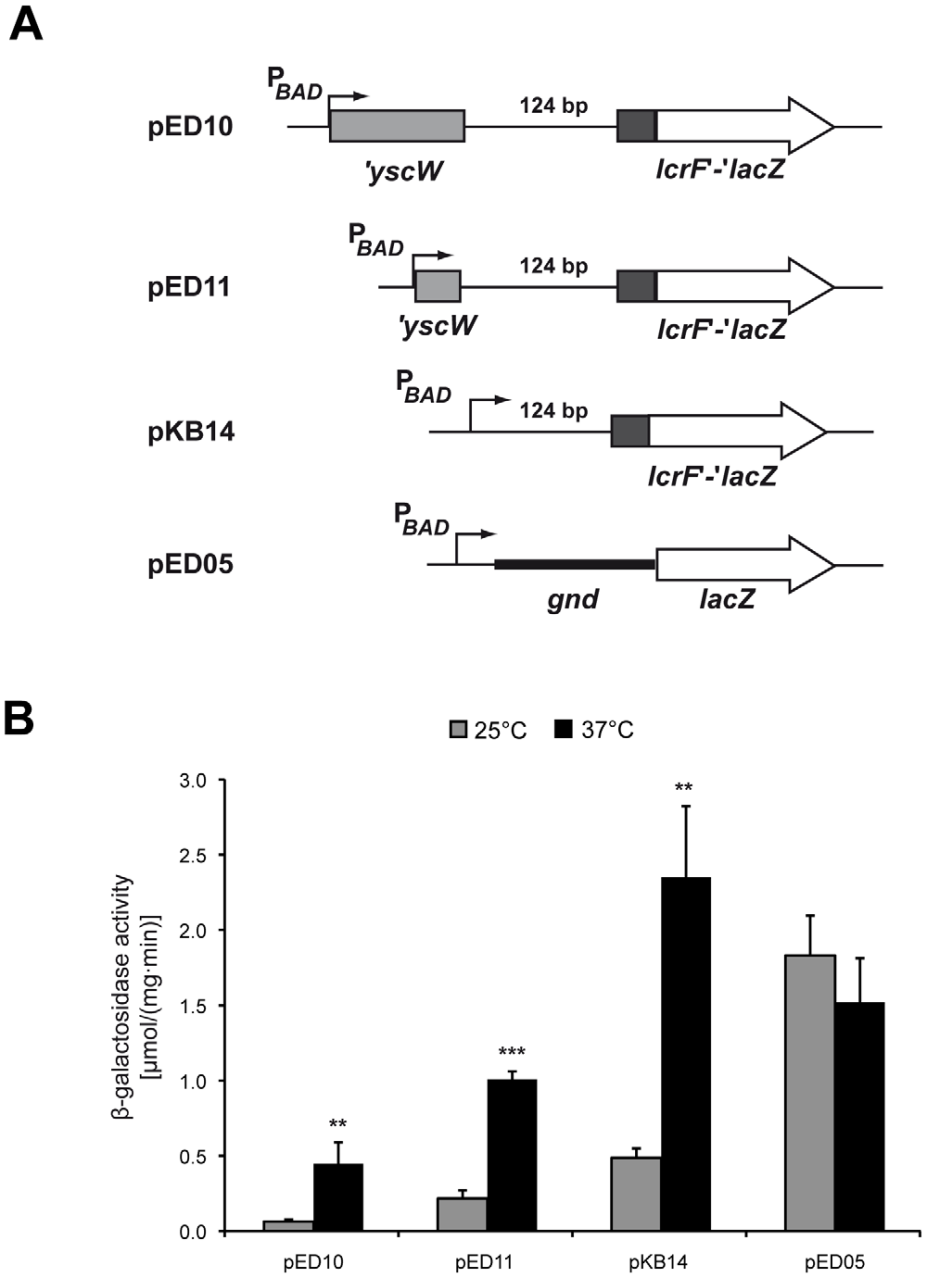
The intergenic region of the *yscW-lcrF* operon is implicated in the temperature control of LcrF production. (**A**) Schematic presentation of the reporter gene fusion harboring the *yscW-lcrF* intergenic region and different portions of *yscW* under control of the P*_BAD_* promoter. (**B**) *E. coli* K-12 harboring the different P*_BAD_*::*yscW-lcrF-lacZ* reporter plasmids (pED10, pED11 and pKB14) or the P*_BAD_*::*gnd-lacZ* control plasmid (pED05) were grown overnight in LB medium at 25°C or 37°C in the presence of 0.05% arabinose. β-Galactosidase activity from overnight cultures was determined and is given in mmol min^−1^ mg^−1^ for comparison. The data represent the average ± SD from at least three different experiments each done in duplicate. Data were analyzed by the Student's t test. Stars indicate the reporter activity that differed significantly between 25°C and 37°C with ** (P<0.01), and *** (P<0.001).

To localize the region responsible for this type of control, we exchanged the *yscW* promoter (P*_yscW_*) against the P*_BAD_* promoter and analyzed *yscW-lcrF-lacZ* expression after induction with 0.05% arabinose at 25°C and 37°C. Thermoregulation was maintained when *yscW-lcrF* was transcribed by P*_BAD_* independent whether the fusion was expressed in *E. coli* or in *Y. pseudotuberculosis* (Figure **4**, **S3**). In contrast, expression of *lacZ* fused to the 5′-UTR of the *Y. pseudotuberculosis* 6-phosphogluconate dehydrogenase gene (*gnd*) in the identical vector system was not affected by the growth temperature. These experiments strongly suggested that the temperature control of *lcrF* expression is mediated by a post-transcriptional mechanism as previously demonstrated in *Y. pestis* [24] and is independent of *Yersinia*-specific factors. Deletions removing different portions of the *yscW* locus or the entire *yscW* gene further demonstrated that presence of the *yscW* gene is dispensable and that the intergenic region of the *yscW-lcrF* operon is sufficient for temperature control of *lcrF* translation (Figure **4** **, S4**).

**Figure 5:**
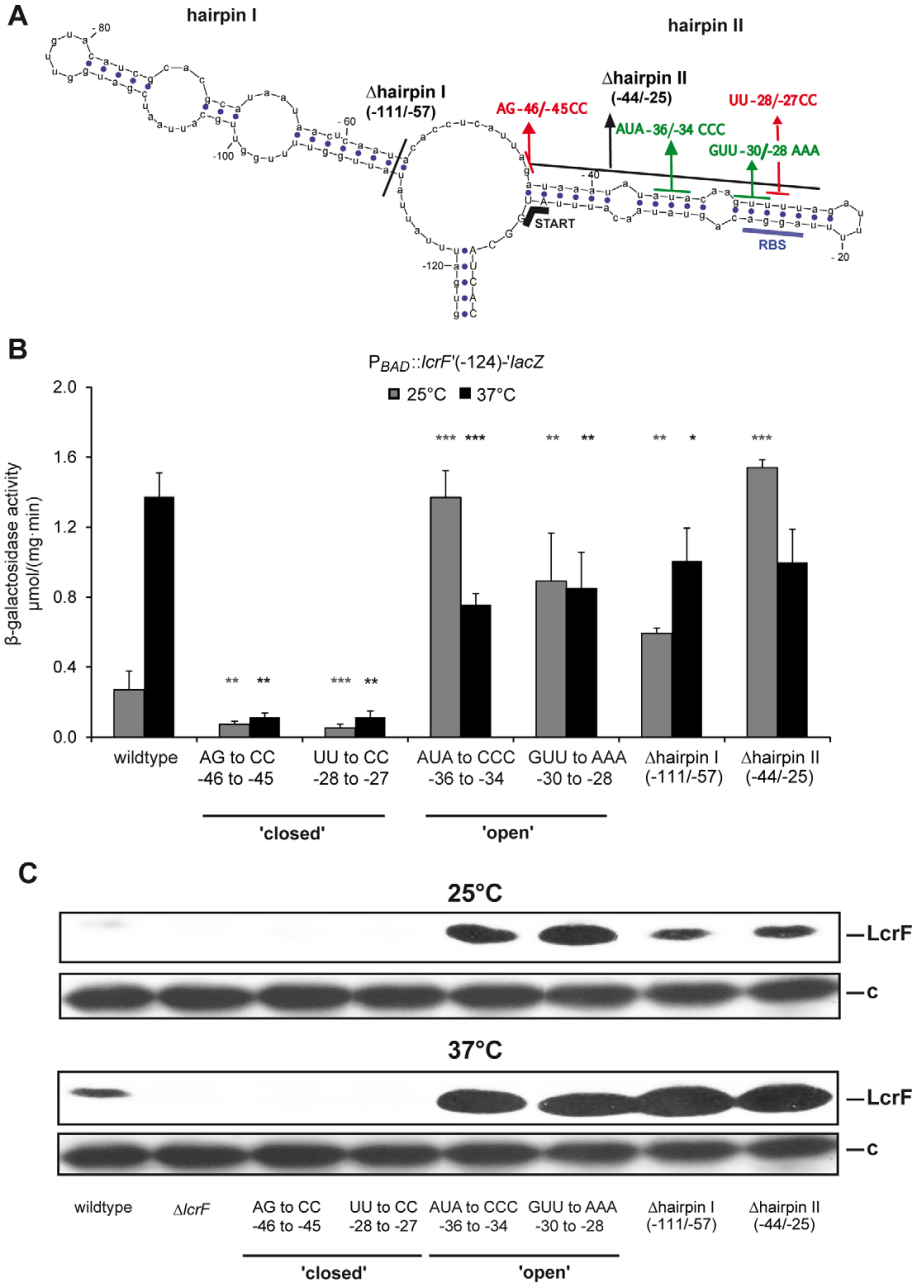
Predicted secondary structure of the *lcrF* RNA thermometer. (**A**) The Mfold program [81] was used for the prediction of the secondary structure of the *yscW-lcrF* 124 nt intergenic region. The most probable prediction with the lowest free energy is shown. The blue dots represent base pairing. The start of the protein synthesis at the AUG start codon (START) and the ribosome binding site (RBS) paired with the fourU motif are labelled. Deletion of the hairpin I and II are indicated. Nucleotide exchanges leading to increased complementarity are marked in red, mutations impairing base pair formation are given in green. Numbers indicate the nucleotides relative to the *lcrF* start codon. (**B**) Strains YPIII harboring the P*_BAD_*::*lcrF*′ (−124)-‘*lacZ*, including the different hairpin deletions or nucleotide exchanges were grown overnight in LB medium at 25°C or 37°C supplemented with 0.05% arabinose. β-Galactosidase activity from overnight cultures was determined and is given in µmol min^−1^ mg^−1^ for comparison. The data represent the average ± SD from at least three different experiments each done in duplicate. Data were analyzed by the Student's t test. Stars indicate the results that differed significantly from those of the wildtype at the same temperature with * (P<0.05), ** (P<0.01), and *** (P<0.001). (**C**) *Y. pseudotuberculosis* strains harboring the deletion and nucleotide substitution illustrated in (**A**) in the pYV were grown at 25°C and 37°C. Whole cell extracts of equal amounts of the bacteria were prepared, separated on a 15% SDS polyacrylamide gel, transferred onto a Immobilon membrane and intracellular LcrF was visualized by Western blotting. A higher molecular weight protein (c) that reacted with the polyclonal antiserum was used as loading control.

Systemic inspection of the 124 nt *yscW-lcrF* intergenic region, comparison with related bacteria (*Y. pestis, Y. enterocolitica*) and secondary structure predictions by Mfold [42] revealed a potential RNA structure composed of two stemloops (hairpin I and II) with a free energy of −19.67 kcal mol^−1^ (Figure **5A**). The first hairpin (57 nt) consists of three base-pairing stretches interrupted by two internal loops and is separated from the second hairpin (hairpin II, 46 nt) by 11 nt. In hairpin II, the ribosomal binding site (RBS) of *lcrF* pairs with a stretch of four uracil residues (fourU) located 26 to 29 nt upstream of the translation initiation site of *lcrF*. This structure resembles a fourU thermometer identified in the 5′-untranslated region of the *Salmonella agsA* gene [43]. Presence of two small loops (C-5/A-6/A-38 and A-12/A-31/A-32) and three imperfect base-pairs in the RBS region (G-15/U-28; G-16/U-27; U-19/G-24) in hairpin II suggested a temperature-labile structure prone to melting at increasing temperatures.

To investigate whether the intergenic region of *yscW-lcrF* forms a functional RNA thermometer we deleted hairpin I (Dhairpin I: −111/−57) or parts of hairpin II (Dhairpin II: −44/−25) and introduced stabilizing (AG-46/-45CC; UU-28/-27CC) and destabilizing (AUA-36/-34CCC; GUU-30/-28AAA) point mutations into in the P*_BAD_*::*lcrF-lacZ* fusion construct and in the *yscW-lcrF* intergenic region of the virulence plasmid pYV (Figure **5A**). Absence of hairpin I (Δhairpin I) resulted in a significant reduction of *lcrF* thermo-induction from 5- to 2-fold (Figure **5B,C**). Expression was already high at 25°C and induction was lost when sequences implicated in the formation of hairpin II (Δhairpin II) were deleted. Similarly, thermo-induced expression of *lcrF* was strongly decreased in both mutations designed to destabilize hairpin II, whereas expression of variants with stabilizing mutations remained repressed upon a temperature upshift and only very small amounts of the LcrF protein were produced at both 25°C and 37°C (Figure **5B,C**). Increase of LcrF levels in the destabilized mutant from 25°C and 37°C demonstrates a two-layer regulation by the thermo-labile YmoA protein and the RNA thermometer. In the following experiments the stabilizing mutation UU-28/-27CC and the destabilizing mutation GUU-30/-28AAA are also referred to as ‘closed’ and ‘open’, respectively. In summary, our data demonstrate that the intergenic region of the *yscW-lcrF* operon contains a thermo-responsive RNA element composed of two hairpins that mediate post-transcriptional control in an RNA thermometer-like manner.

### Temperature-dependent structural changes of the intergenic, non-translated *yscW-lcrF* mRNA

**Figure 6:**
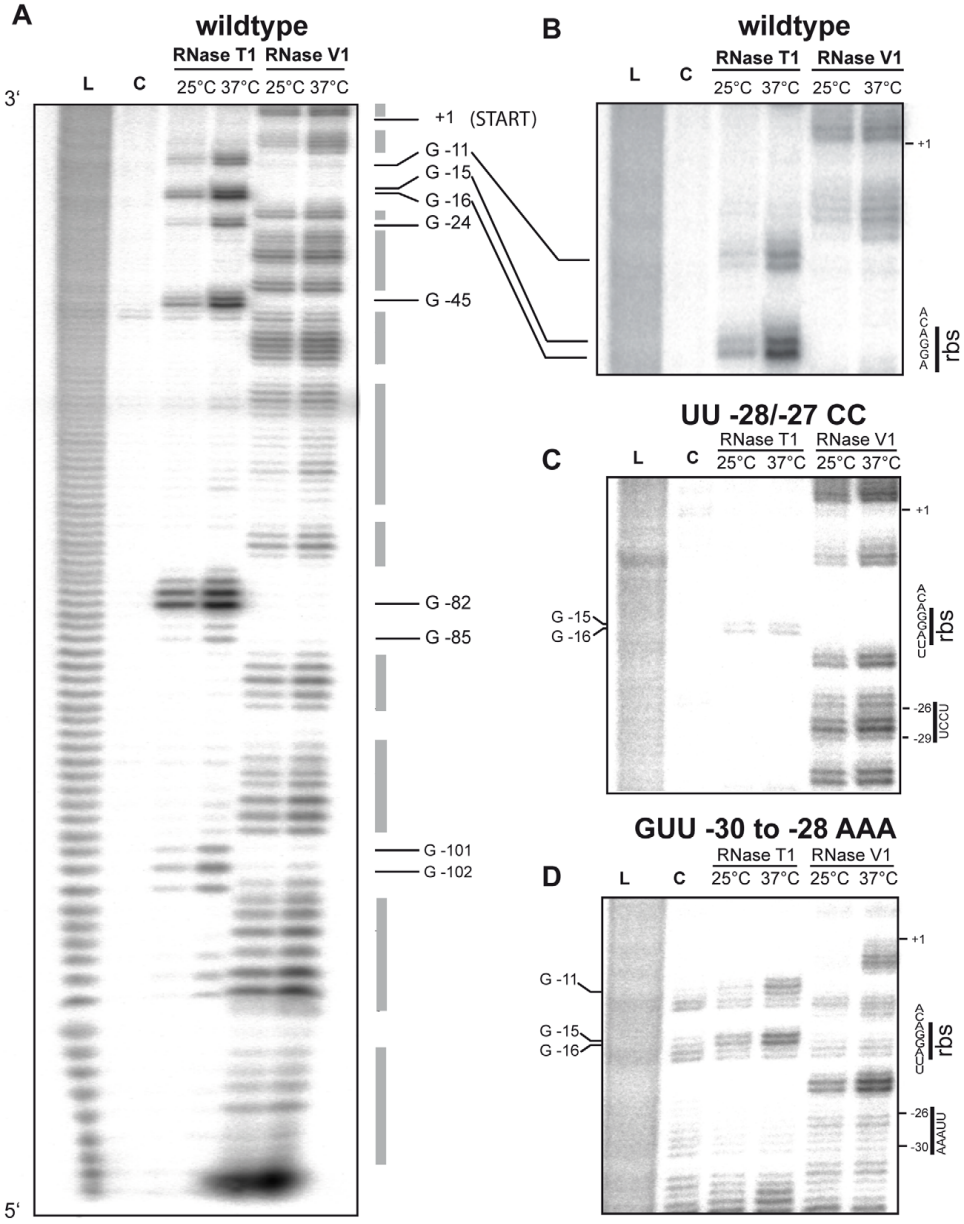
Enzymatic probing of the *yscW-lcrF* intergenic region variants. (**A**) Enzymatic hydrolysis of the *yscW-lcrF* wildtype sequence with endonucleases T1 (0.001 U/µl) and V (0.0002 U/µl) performed on the 5′-end labelled intergenic region between the *yscW* and *lcrF* gene on the pYV at 25°C and 37°C. The G nucleotides in single stranded regions are indicated. (**B**) Magnification of the enzymatic probing pattern of the fourU/Shine-Dalgarno region. (**C**) Enzymatic probing of the *yscW-lcrF* of the repressed UU-28/-27CC variant, and (**D**) enzymatic probing of the *yscW-lcrF* of the derepressed GUU-30/-28AAA variant. The RNA fragments were separated on 8% polyacrylamide gels. Lane L: alkaline ladder; lane C: controls without RNase. The Shine-Dalgarno sequence and the nucleotide exchanges are indicated.

In order to examine the architecture of the predicted RNA structure experimentally, we determined the structure and the nature of thermo-induced conformational changes of the intergenic *yscW-lcrF* mRNA by enzymatic probing at 25°C and 37°C using RNAse T1 (cleaves 3′ of unpaired guanines) and double-strand specific RNase V1. Due to the large size of the full-length *yscW-lcrF* transcript, the structure of a shorter RNA fragment including the entire *yscW-lcrF* intergenic region (5′-UTR of *lcrF*) was probed (Figure **6A, B**). The cleavage pattern at 25°C was in full agreement with the predicted two hairpin structure (Figure **5A**). RNase T1 digestion at positions −81 to −85 and positions −101 to −102 as well as the sensitivity of adjacent regions to RNase V1 cleavage (positions −71 to −68; −78 to −75; −87 to −90; −94 to −99; −103 to −108) confirmed the predicted secondary structure of hairpin I containing three loop segments. Also hairpin II seems to form the predicted structure (protection to RNase V1 at positions −24 and −20). Consistent with its function as a temperature sensor, this stemloop is more dynamic and adapts a thermo-sensitive conformation that seems to open after a shift to 37°C. As shown in Figure **6**, the stem region including the imperfect UUUU/AGGA base pairs with the RBS and flanking regions is more resistant to RNases T1 at 25°C than at 37°C. Temperature-induced melting of the stemloop II at 37°C is also supported by digest with the RNase V1, which is less active at 25°C. To confirm that RNase T1 cleavage at the RBS is the result of structural changes and not the result of an induced activity of RNase T1 at higher temperatures, we quantified the intensities of the T1 cleavage sites at 25°C and 37°C using the AlphaEaseFC program (Cell Biosciences, USA). The cleavage intensity at 37°C relative to 25°C was 4-fold at G15/G16 within the RBS, compared to 1.3-fold at G82 in hairpin I, and 1.7- to 2.3-fold at adjacent T1 cleavage sites (G11, G24, G45) in hairpin II. These results indicated that the RBS within hairpin II represents the primary temperature-sensing site within the *lcrF* RNA thermometer. We also performed enzymatic probing with the *yscW-lcrF* mRNA derivatives including the stabilizing and destabilizing nucleotide exchanges in hairpin II. Analysis of the UU-28/-27CC mutation indicated the generation of a thermostable stemloop II structure as neither the RBS nor the anti-RBS fourU sequence was accessible to RNases T1 at 25°C and 37°C (Figure **6C**). Complete protection of the ribosomal binding site is in full agreement with reduced expression of *lcrF* (Figure **5B**). In contrast, introduction of the derepressing GUU-30/-28AAA exchanges resulted in an altered, less stable structure in which the RBS sequence is more accessible at 25°C and 37°C (Figure **6D**).

### Ribosomes bind to the *lcrF* transcript at 37°C but not at 25°C

**Figure 7:**
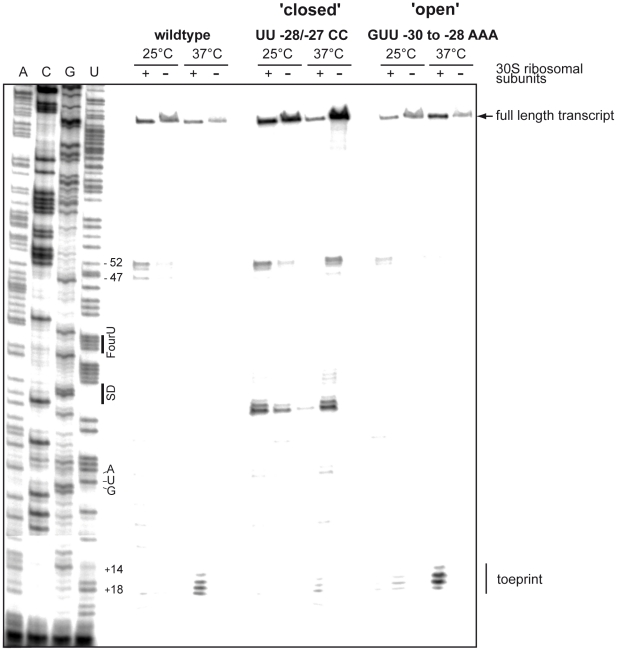
Temperature-dependent binding of ribosomes to the *yscW-lcrF* intergenic region. Toeprinting analysis was performed with the wildtype, the repressed (UU-28/-27CC) and derepressed (GUU-30/-28AAA) variants as described in material and methods. The presence (+) and absence (−) of the 30S ribosomal subunits are indicated. The terminated primer extension products (toeprints) are marked. The sequencing ladder (ACGU) generated with the same *lcrF*-specific primer is loaded on the left. The positions of the fourU motif, the Shine-Dalgarno sequence and the start codon AUG are indicated.

To demonstrate temperature-dependent interaction of the 30S ribosome with the RBS in the intergenic region of the *yscW-lcrF* mRNA, we performed toeprinting analysis. Ribosomal subunits and the initiator tRNA^fMet^ were added after annealing of the *lcrF* specific reverse primer to the *yscW-lcrF* template and incubated at 25°C or 37°C. The primer extension reaction was not inhibited at 25°C and/or in the absence of the 30S ribosome. However, at 37°C a toeprint (prematurely terminated product) was detected at position +14/+18 relative to the translational start site, demonstrating the formation of a ternary translation initiation complex composed of the *yscW-lcrF* mRNA, the 30S ribosome and tRNA^fMet^ (Figure **7**). More prominent toeprint signals were observed when the destabilizing GUU-30/-28AAA exchanges were introduced, whereas significantly read-through up to the full length transcript and less toeprint signals were found with the stabilizing UU-28/-27CC variant (Figure **7**). Taken together, this experiment showed that a thermo-induced interaction of the ribosome with the *lcrF* translation initiation site is facilitated at body temperature and occurs in the absence of any other bacterial factors.

### The *lcrF* RNA thermometer is crucial for virulence

**Figure 8:**
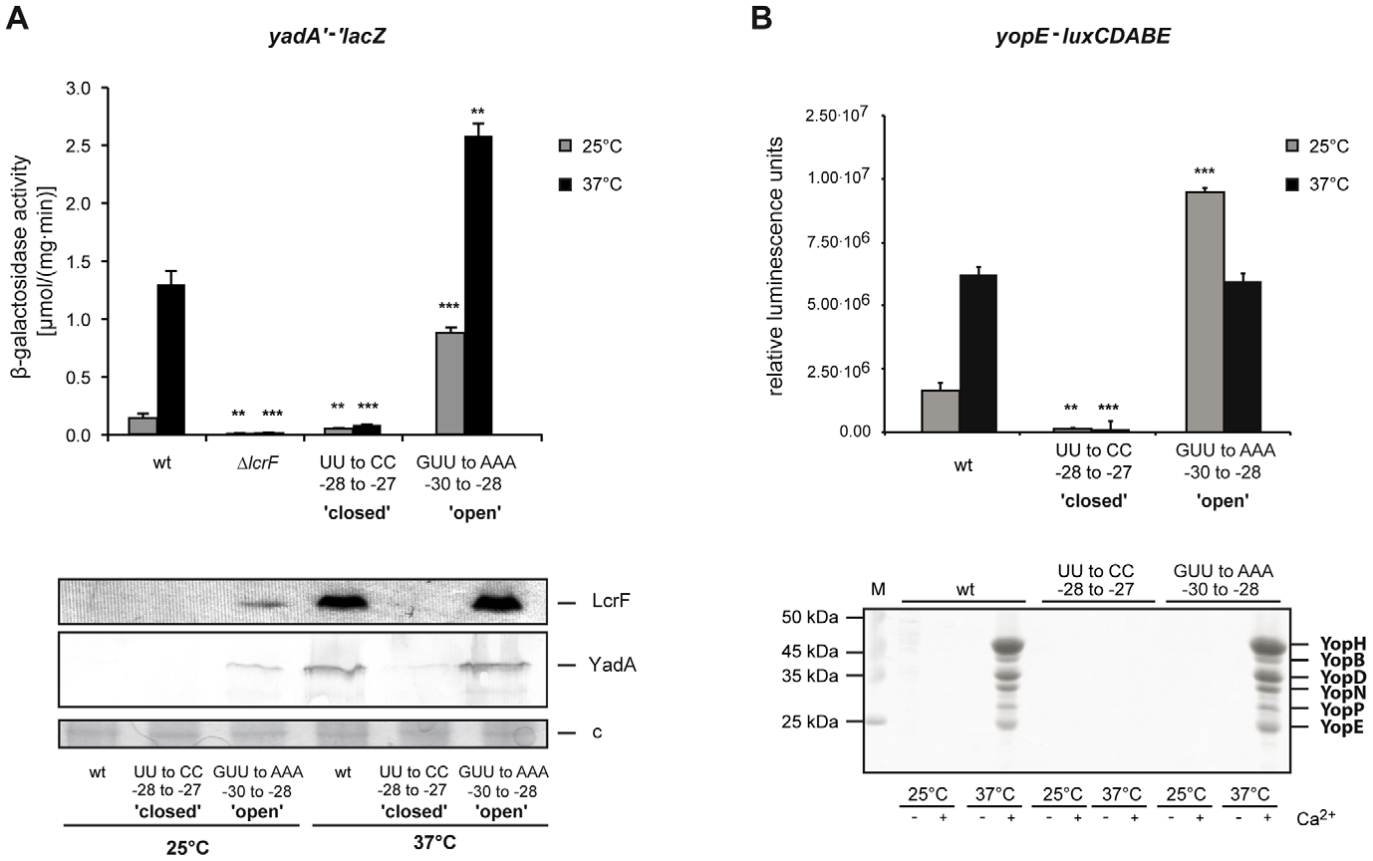
*lcrF* thermosensor-dependent expression of the *yadA* and *yopE* genes. (**A**) Strains YPIII (wildtype), YP66 (Δ*lcrF*) and the repressed and derepressed *yscW-lcrF* variants YP90 (UU-28/-27CC) and YP95 (GUU-30/-28AAA) harboring the *yadA-lacZ* fusion plasmid pSF1 were grown in LB medium at 25°C or 37°C. β-Galactosidase activity from overnight cultures was determined and is given in µmol min^−1^ mg^−1^ for comparison. The data represent the average ± SD from at least three different experiments each done in duplicate. Data were analyzed by the Student's t test. Stars indicate the results that differed significantly from those of the wildtype at the same temperature with ** (P<0.01), and *** (P<0.001). Whole-cell extracts from overnight cultures of *Y. pseudotuberculosis* YPIII (wildtype) and the repressed and derepressed *yscW-lcrF* variants YP90 (UU-28/-27CC) and YP95 (GUU-30/-28AAA) grown at 25°C or 37°C were prepared, and analysed by Western blotting with a polyclonal antibody directed against LcrF and YadA. A higher molecular weight protein (c) was used as control the protein content of the cell extracts. (**B**) Strains YPIII (wildtype) and the repressed and derepressed *yscW-lcrF* variants YP90 (UU-28/-27CC) and YP95 (GUU-30/-28AAA) harboring the *yopE-luxCDABE* plasmid pWO34 were grown in LB medium at 25°C and 37°C. Bioluminescence emitted by the cultures was monitored and is given as relative luminescence units (RLU) and represents the mean of three independent experiments done in triplicate. Data were analyzed by the Student's t test. Stars indicate the results that differed significantly from those of the wildtype at the same temperature with ** (P<0.01), and *** (P<0.001). The panel below shows TCA-precipitated supernatants of YPIII (wildtype), the repressed and derepressed *yscW-lcrF* variants YP90 (UU-28/-27CC) and YP95 (GUU-30/-28AAA) grown at 25°C and 37°C in the presence (+) or absence (−) of Ca^2+^. The secreted Yop proteins are indicated.

To analyze whether this mechanism of post-transcriptional thermoregulation has an important impact on virulence, we first tested whether introduction of the ‘open’ (GUU-30/-28AAA) and ‘closed’ (UU-28/-27CC) mutations into the *yscW-lcrF* intergenic region resulted in mis-regulation of the LcrF-dependent virulence genes *yadA,* and *yopE* encoded on the *Yersinia* virulence plasmid pYV (Figure **8**). Consistent with previous results, *yadA* and *yopE* transcription as well as YadA synthesis was temperature-induced in the wildtype. Expression was abolished in mutants with stabilizing nucleotide exchanges in the 5′-UTR of *lcrF*. In contrast, destabilizing substitutions led to increased *yadA* and *yopE* expression already at 25°C. The LcrF-dependent *yadA-lacZ* expression and LcrF synthesis increased at 37°C in the presence of the ‘open’ mutation (Figure **5**, **8**) which can be explained by the loss of YmoA-dependent control of *lcrF* expression.

*In vitro*, Yop secretion is generally blocked in the presence of millimolar amounts of extracellular Ca^2+^ but it can be induced upon Ca^2+^-complexation with sodium oxalate (Na_2_C_2_O_4_) [44]–[46]. As expected, concentration of secreted Yop proteins by the wildtype (YPIII) and the ‘open’ strain YP95 (GUU-30/-28AAA) was high at 37°C in the absence of Ca^2+^, but no Yops could be detected in the supernatants of the ‘closed’ strain YP90 (UU-28/-27CC) under the same growth conditions. Strikingly, although LcrF synthesis and the LcrF-dependent *yopE* gene expression are already induced in the derepressed strain YP95 (GUU-30/-28AAA) at 25°C, no Yop secretion was detectable after Ca^2+^ depletion, indicating that a temperature-dependent mechanism blocks YopE production and/or secretion at low temperatures.

**Figure 9:**
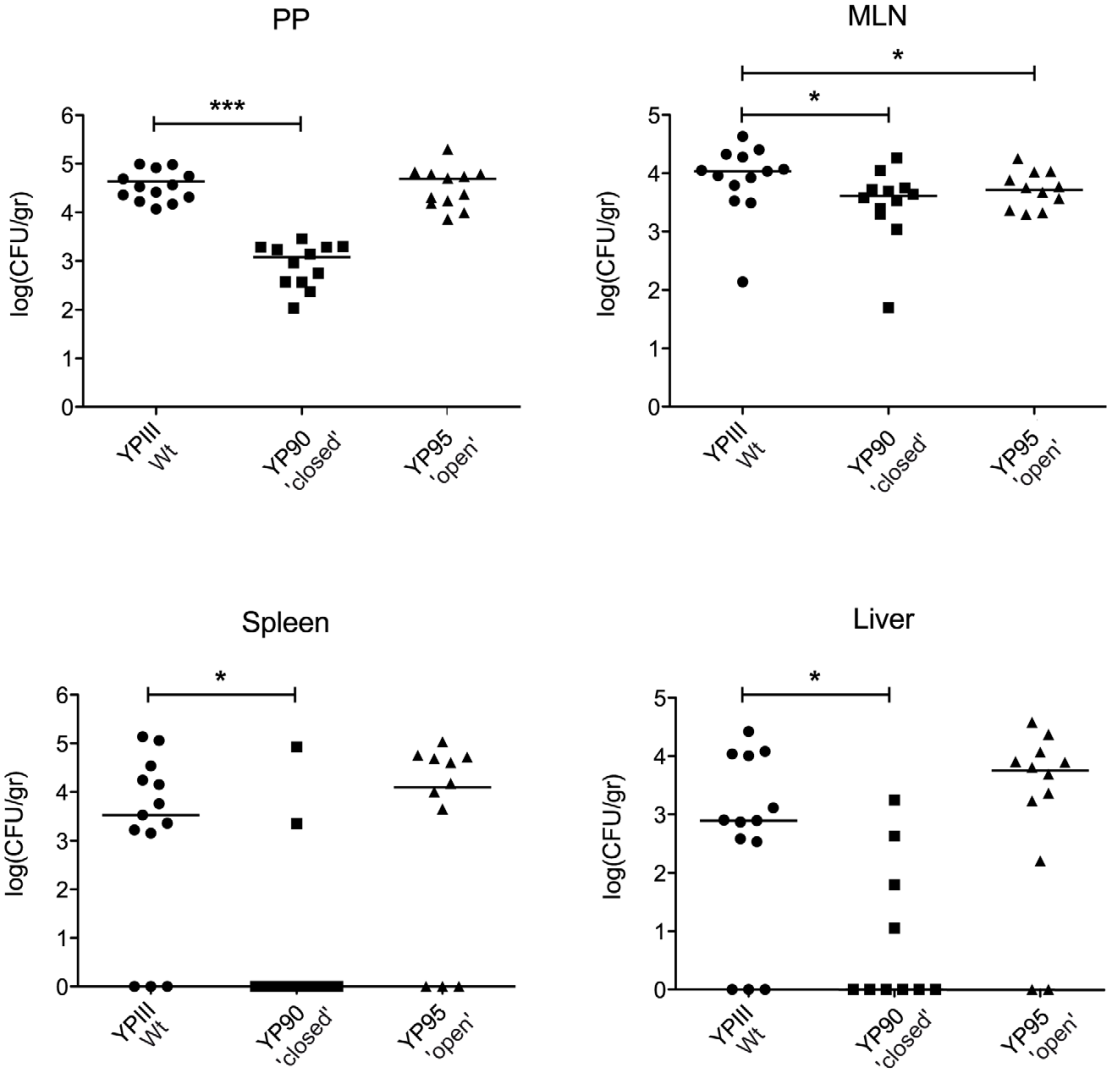
Influence of the *lcrF* RNA thermometer on tissue colonization by *Y. pseudotuberculosis*. Strains YPIII (wildtype) and the *yscW-lcrF* variants YP90 (UU-28/-27CC) and YP95 (GUU-30/-28AAA) were infected intragastrically (5·10^8^ CFU/mice) into BALB/c mice (n = 12/strain). After three days of infection, mice were sacrificed and the number of bacteria in homogenized host tissues and organs was determined by plating. Solid lines indicate the means. The statistical significances between the wildtype and the repressed and derepressed *lcrF* RNA thermometer variants were determined by Student's t test. P-values: *: <0.05; ***: <0.001.

In order to define the influence of the *lcrF* RNA thermometer on bacterial pathogenesis, we compared survival and dissemination of the *Y. pseudotuberculosis* wildtype YPIII and the repressed and derepressed mutant strains YP90 (UU-28/-27CC) and YP95 (GUU-30/-28AAA) in the mouse model. Presence of each strain was examined three days after intragastrically infection of a group of BALB/c mice (*n* = 12) with 5·10^8^ bacteria by quantifying the number of bacteria that reached and survived in the Peyer's patches (PP), the mesenterial lymph nodes (MLN), liver and spleen. Significantly reduced numbers of the repressed YP90 (UU-28/-27CC) mutant strain were recovered from the Peyer's patches and organs (Figure **9**), very similar to the *lcrF* mutant strain YP66 (Figure **S5**). We also introduced the ‘closed’ and ‘open’ mutation into the more virulent *Y. pseudotuberculosis* strain IP32953. Oral infections with IP32953 and the isogenic ‘open’ variant (YPIP02) led to a higher organ burden three days post infection. However, the number of bacteria was similarly reduced in the host tissues with the ‘closed’ mutant (Figure **S6**). This demonstrated that a repression of the fourU RNA thermometer reduced virulence and showed that the structural rearrangements of the 5′-UTR of the *lcrF* mRNA affects pathogenesis of both *Yersinia* strains. Our results also showed that introduction of derepressing nucleotide exchanges (GUU-30/-28AAA) had no or only a minor effect on the colonisation of host tissue (Figure **9**, **S6**).

**Figure 10:**
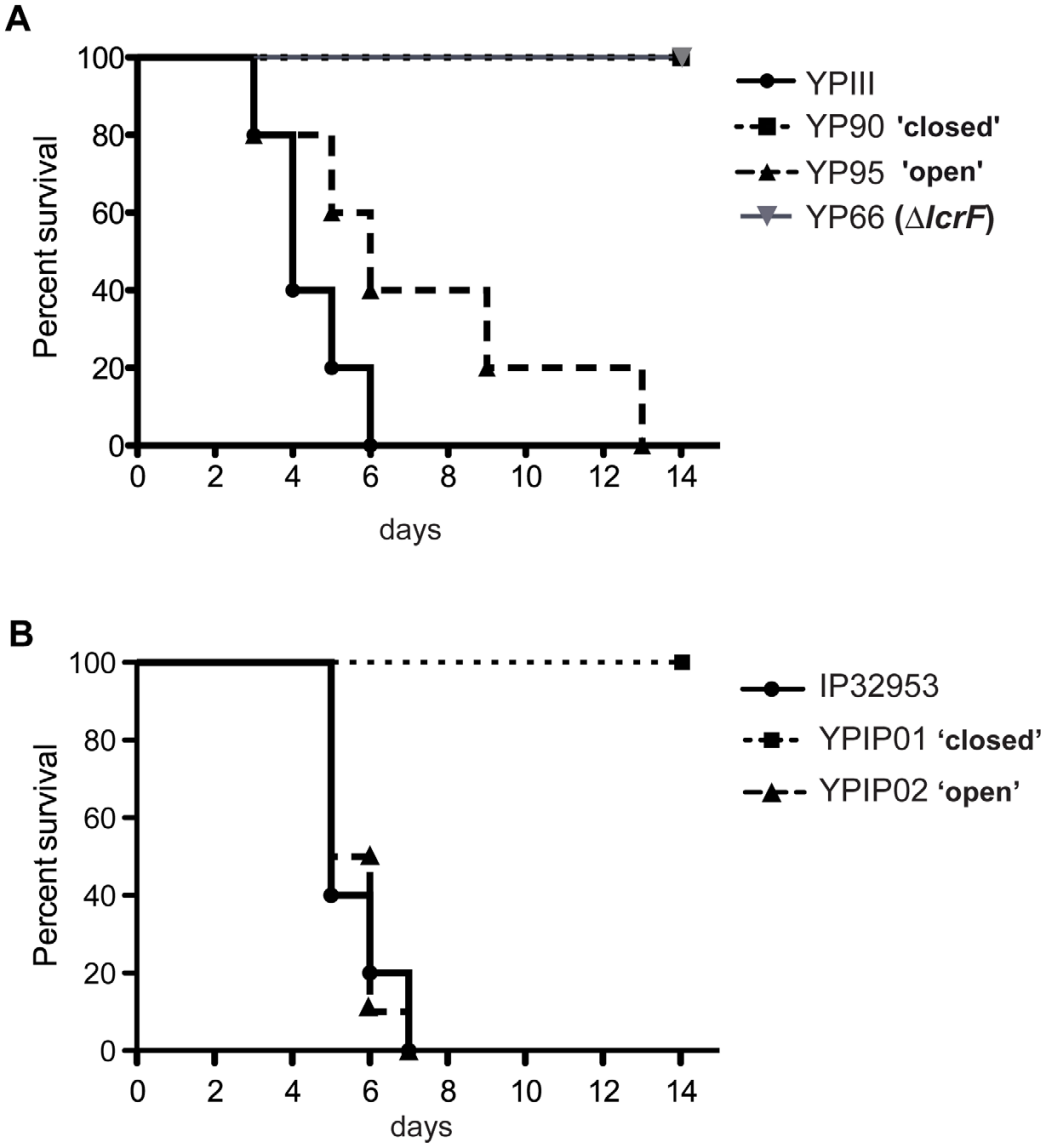
*lcrF* RNA thermometer variants affect survival of *Y. pseudotuberculosis* infected mice. (**A**) 2·10^9^ CFU of *Y. pseudotuberculosis* YPIII (wildtype), the *yscW-lcrF* variants YP90 (UU-28/-27CC) and YP95 (GUU-30/-28AAA), and YP66 (Δ*lcrF*) were used to orally infect BALB/c mice (n = 10/strain). (**B**) 1·10^10^ CFU of *Y. pseudotuberculosis* IP32953 (wildtype), the *yscW-lcrF* variants YPIP01 (UU-28/-27CC) and YPIP02 (GUU-30/-28AAA) were used to orally infect BALB/c mice (n = 10/strain). Survival of the mice was monitored up to 14 days.

To complement the infection experiments, the potential of the different *lcrF* RNA thermometer mutant strains to cause a lethal infection was determined. Groups of BALB/c mice (*n* = 10) were infected intragastrically with 2·10^9^ bacteria of each mutant, YP90 or YPIP01 (UU-28/-27CC) and YP95 or YPIP02 (GUU-30/-28AAA) and the wildtype strains YPIII or IP32953. Survival of the mice was followed over 14 days and date of death was recorded (Figure **10**). All mice infected with the wildtype strain showed visible signs of infection by day three post infection (e.g. lethargy, rough fur) and succumbed to infection between day three to six post challenge. Strikingly, none of the mice infected with the repressed mutant strain YP90 or YPIP01 (UU-28/-27CC) developed disease symptoms and all were still alive 14 days after infection, similar to the Δ*lcrF* mutant strain YP66 (Figure **10**). This indicated that stabilization of hairpin II renders the bacteria avirulent. In contrast, the destabilizing mutations in the *lcrF* RNA thermometer had no apparent effect on the initial rate of death, and did not cause a higher mortality than the wildtype over a 14-day period (Figure **10**). The average day to death of mice challenged with the “open” mutant variants YP95 or YPIP02 (GUU-30/-28AAA) was similar or increased from four to seven days. Taken together, this illustrates that the RNA thermometer is crucial for virulence, as it plays an important role adjusting the appropriate amounts of LcrF for maximal pathogenicity.

## Discussion

Many environmental signals are sensed by enteric pathogens such as *Y. pseudotuberculosis* in order to induce and adjust expression of virulence factors upon host entry and during ongoing infections. Temperature is among the most important decisive parameters for an intestinal pathogen, indicating that it successfully invaded a warm-blooded host. A prerequisite for an appropriate response to temperature changes is precise thermosensing, and different principles governing the temperature-sensing mechanism have been uncovered for a variety of macromolecules [47]–[48]. Thermo-induced structural changes in supercoiled or intrinsically curved DNA have long been known to manipulate gene expression by altering the accessibility of promoter elements [49]–[50]. Recently, also regulatory proteins were shown to act as intrinsic thermosensors to adjust their DNA-binding properties [8], [51]–[53], and experimental evidence accumulated that also RNA plays a fundamental role in temperature sensing [54]–[56].

**Figure 11:**
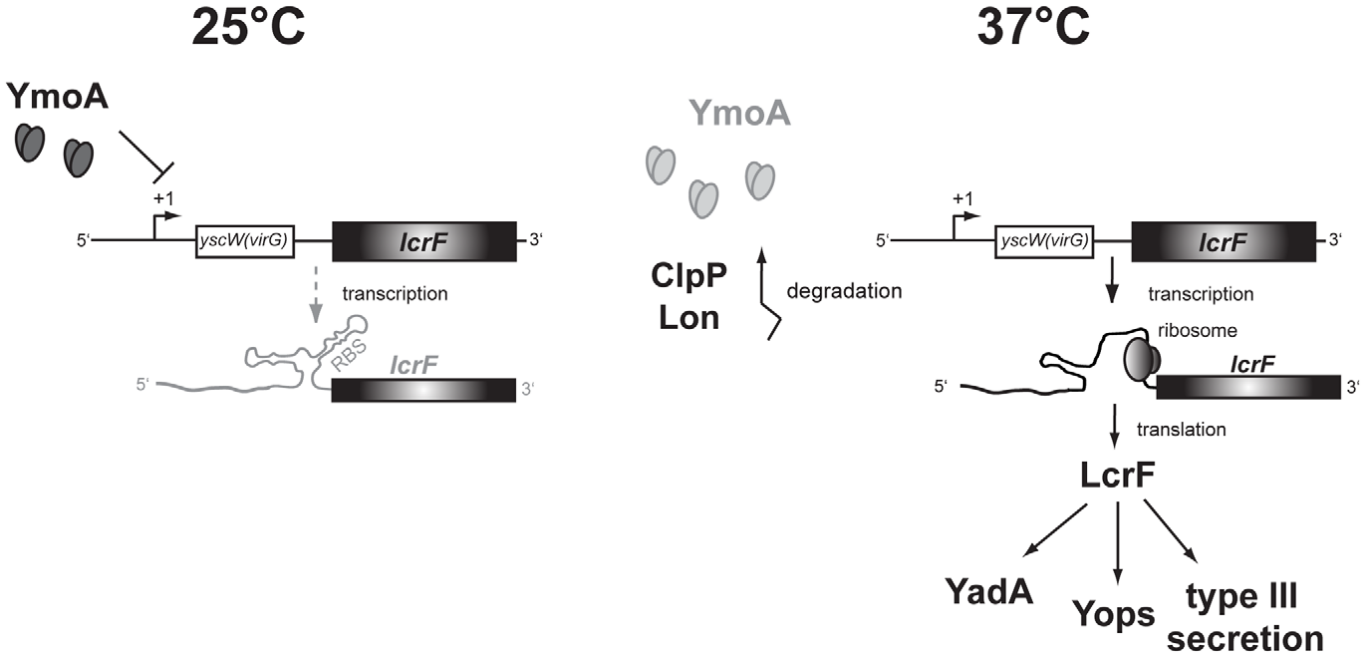
Model of thermoregulated expression of LcrF synthesis. At moderate growth temperature, transcription of the *yscW-lcrF* operon is repressed by the regulatory protein YmoA through sequences located downstream of the transcription initiation site. In addition, translation of the *lcrF* transcript is blocked through the formation of a two-stemloop structure within the intergenic region which sequesters the RBS and prevents access of the ribosomes. After a sudden temperature upshift upon host entry, YmoA is rapidly degraded by the ClpP and Lon proteases, leading to an enhanced transcription of the *yscW-lcrF* operon. Furthermore, thermally-induced conformational changes allow access of ribosomes and translation of the *lcrF* transcript leading to LcrF synthesis and induction of all LcrF-dependent virulence genes of *Yersinia*.

Although control of translation initiation by limiting the access to the ribosome-binding site has been reported earlier, the full dimension to which structured mRNAs contribute to thermosensing has only recently been recognized. Several distinct and structurally unrelated RNA sensors have been identified in bacteria, but almost all control the synthesis of heat shock proteins. To our knowledge only one RNA thermometer located upstream of the virulence regulator gene *prfA* of *Listeria monocytogenes* has been described to regulate virulence gene expression and invasion into cultured cells [57]. However, its impact for pathogenesis, e.g. initiation or progression of the infection has not been investigated. In this study, we report the existence of an unusual intergenic, two-stemloop RNA thermometer and provide first experimental evidence that its function is crucial for *Y. pseudotuberculosis* virulence in a mouse model. Two temperature-sensing modules, the thermo-sensitive virulence modulator protein YmoA and the RNA thermosensor, act in concert to optimize temperature perception and fine-tune virulence gene expression during infection (Figure **11**).

A comprehensive expression analysis revealed, that the *lcrF* gene is transcribed from a single σ^70^-specific promoter of the *yscW* gene (formerly named *virG*) which is located 124 bp upstream of *lcrF*. Cotranscription is consistent with the observation that the *yscW-lcrF* locus is similar to the last two genes of the *exsC-exsB-exsA* operon of *Pseudomonas aeruginosa* required for the ExoS effector synthesis [58]. It also reconciles previous contradictory models for temperature control of *lcrF* (*virF*) expression in *Y. pestis* and *Y. enterocolitica*. Cornelis *et al.* showed that *virF* of *Y. enterocolitica* itself is thermoregulated at the transcriptional level [44]. In that study, *virF::cat* fusions and *virF* Northern blots demonstrated transcription activation at elevated temperature. Based on the present analysis, thermo-dependent *virF* expression can be explained by YmoA-dependent repression of the *yscW* promoter that is eliminated at higher temperatures due to increased degradation of YmoA by the Lon and Clp proteases [37].

In contrast, *lcrF-lacZ* reporter fusions in *Y. pestis,* including only 206 bp upstream of the *lcrF* start codon, were found to be insensitive to temperature changes, although much higher levels of the LcrF protein were produced in *Y. pestis* with raising temperature [24], [27]. This implied that a different post-transcriptional mechanism modulates LcrF levels in response to temperature in this organism. A simple model for *lcrF* thermal regulation has been suggested in which a predicted thermo-labile stem-loop (identical to the upper part of hairpin II) sequesters the *lcrF* ribosomal binding site [27], [43], but its function has never been proven.

Reporter gene assays and a detailed structure-function analysis of the isolated intergenic region in this study provide evidence for a functional RNA thermometer in which temperature regulation of *lcrF* is mediated in the absence of the natural promoter. Structural probing experiments demonstrated the formation of two hairpins of which hairpin II includes a consecutive stretch of four uridine nucleotides (fourU motif), which base pair with the RBS, and two internal unpaired bulges. Mutational analyses and toeprinting experiments further showed that this RNA structure is sufficiently stable to resist melting at moderate temperature (25°C), but it allows partial unfolding at body temperature (37°C) which permits access of ribosomes and initiation of *lcrF* translation. Hairpin I was not essential for thermosensing, but it seems to support proper folding and/or the stability of the ‘closed’ RNA thermometer structure, as generally higher amounts of the LcrF protein were detectable in Δloop1 mutant variants. Importance of this RNA structure is further supported by the fact that the RNA thermosensor sequence is 100% identical in all human pathogenic *Yersinia* species, although the homology between *Y. pseudotuberculosis* and *Y. enterocolitica* is less than 70% and several nucleotide substitutions are detectable in the adjacent *yscW* and *lcrF* genes (Figure **S7**). In fact, a P*_BAD_*::*lcrF-lacZ* reporter in *Y. enterocolitica* 8081 exhibited a similar thermo-dependent expression pattern, indicating that the RNA thermometer is also functional in this *Yersinia* species (R. Steinmann, K. Böhme, unpublished results).

The intergenic position of the *Yersinia* RNA thermosensor is unique. All previously known RNA thermometers are positioned at the 5′-end of heat shock or virulence transcripts [54]. Also sequence and structure of the *Yersinia* thermometer deviate significantly from the thermosensor controlling virulence genes of *L. monocytogenes.* The listerial RNA thermometer is positioned within the untranslated region (5′-UTR) of the *prfA* mRNA and forms one extended stemloop structure (130 nt) in which the ribosome binding site and the start codon locate in two small and unpaired bulges within the long hairpin structure. This overall structure prevents translation at moderate temperature but is destabilized at 37°C through additional melting of the loops facilitating the access of ribosomes [57].

The hairpin II of the *lcrF* 5′-UTR bears highest resemblance with fourU elements (UUUU pairing with AGGA) predicted in the 5′-UTR of the heat shock genes *groES* and *dnaJ* of *Staphylococcus aureus* and *Brucella melitensis,* and *agsA* of *Salmonella enterica* serovar Typhimurium [43]. Only the *agsA* leader sequence has been studied in detail. It is short (58 nt), simply structured and folds into two hairpins. Hairpin II with the fourU region and an unpaired internal G-A loop was temperature-responsive and melted at heat shock temperature while hairpin I remained stable [43]. A stabilizing G-C pair in close vicinity to the fourU motif and Mg^2+^ ions are required to set the melting temperature to heat shock conditions [59], [60]. Hairpin II of the *Yersinia* thermometer is devoid of the stabilizing G-C pair, and contains a large number of weak A–U and G–U base pairs interrupted by two asymmetric internal loops (Figure **5A**). These features might contribute to setting the melting temperature to a more moderate temperature provided by the mammalian host.

The *Listeria prfA* and the fourU elements are clearly distinct from the complex structured thermometer embedded in the coding region of the *rpoH* gene of *E. coli* [61] and the widespread class of ROSE-type thermometers, a conserved regulatory element found in the 5′-UTR of heat shock genes in many α- and γ-proteobacteria [54]–[55]. ROSE elements range from 60 to 110 nt and form a complex secondary structure of 2–4 hairpins, of which the 5′-located stemloop(s) remain folded whereas the 3′-proximal hairpin including the ribosome binding site is thermo-labile and melts upon heat shock. The ROSE class of thermometers contains a UUGCU/AGGA motif in which the highly conserved 5′-G residue pairs in a *syn-anti* conformation with the second G in the AGGA stretch of the ribosome binding site followed by non-canonical interactions including a triple UC-U and a U-U pair [62].

The structure and thermo-induced conformational changes have been studied with several prototypic RNA thermometers. However, the physiological relevance, e.g. for heat resistance and for recovery in the post-stress situation, has only been proven for the *Syncheocystis hsp17* thermometer [63]. Here we provide first experimental evidence that a functional *lcrF* RNA thermometer is crucial for *Yersinia* pathogenesis. A repressing ‘closed’ mutation resulted in a strong reduction of the bacterial burden in the PP, MLN, liver and spleen, and afforded a dramatic survival advantage, similar to *lcrF*-deficient strains. Presence of the *Yersinia* LcrF protein is very critical for virulence as it controls production of the well-characterized virulence determinants, the antiphagocytic Yop effectors and their type III secretion machinery. Evidence of the importance of this protective immune defense strategy derives from pYV-cured strains, which rendered the bacteria completely avirulent, and from studies with *Yersinia lcrF* knock-out strains which were severely attenuated in mouse models of septic, oral and nasal infection [10], [64]–[65].

Another interesting aspect is that *Yersinia* does not profit from elevated LcrF levels provided by a destabilized RNA structure during infection. Strains carrying the ‘open’ *lcrF* variant are attenuated or exhibit the virulence potential of the wildtype strain. Likewise, overproduction of Hsp17 by an ‘open’ thermometer in *Syncheocystis* provided a burden to photosynthetic performance and bacterial fitness [63]. It is very likely that additional control mechanisms prevent Yop production and/or secretion when not needed to maintain maximal bacterial fitness. Although significantly higher levels of the LcrF protein were produced in the ‘open’ UU-28/-27CC mutation at 25°C Yop proteins were not detectable in the supernatants. This is consistent with a previous study demonstrating that LcrF(VirF) overexpression in *Y. enterocolitica* under the control of the *tac* promoter did not result in Yop secretion at 25°C [31]. This strongly indicates that low temperature not only prevents the opening of the *lcrF* RNA thermometer, but also impedes Yop secretion which might be explained by the fact that some T3S genes are only activated by temperature and not by LcrF [31].

Similar to other RNA thermometers, the *yscW-lcrF* intergenic region accounts only in part for the drastic induction of LcrF after temperature upshift. Apparently, very rapid and efficient activation of LcrF production is achieved by the combination of separate regulatory modules. Thermal induction of LcrF translation mediated by the RNA thermometer is combined with temperature-regulated proteolysis of YmoA repressing *yscW-lcrF* expression. Regulatory cascades composed of an alternative sigma factor and an RNA thermometer have been reported [55], [63], [66]. They might be able to integrate several independent signals (e.g. heat and unfolded proteins), whereas the two-layered control by a thermo-sensitive regulator and an RNA thermometer discovered in *Yersinia* presents a novel strategy to strictly adjust virulence gene expression to the presence in the warm-blooded host.

Both thermosensing mechanisms appear to be complemented by yet another control level. The *yscW-lcrF* RNA is highly unstable in *Y. pseudotuberculosis*, which is consistent with a previous study reporting that *lcrF* mRNA degradation was so fast in *Y. pestis* that the transcript could not reliably be detected [24]. Recently, it has been shown that the translocator pore protein YopD recognizes the 5′-ends of transcripts of all type III secretion genes and facilitates their degradation [67]. A negative YopD effect was also observed for LcrF synthesis (R. Steinmann, unpublished results) which could be explained by YopD-mediated stabilization of hairpin II or YopD-induced degradation of the *yscW-lcrF* transcript. Translocation of the YopD protein upon host cell contact would then result in stabilization and increased translation of the LcrF virulence activator, adjusting injectisome and Yop production according to effector translocation.

Presence of highly homologous 5′-UTRs and structurally conserved RNA thermometers in the *yscW-lcrF* intergenic region of all pathogenic *Yersinia* species suggests that they are highly important to adjust biological fitness and virulence. RNA-based regulation of LcrF synthesis is very rapid, highly efficient and less energy-consumptive as it allows fast induction upon host entry and tissue contact, and permits immediate shut-off when the initiating signals are removed. This is particularly important as failure to perform type III secretion results in avirulence due to rapid clearance by the host. On the other hand, uncontrolled secretion of Yops was found to be highly detrimental and cause a severe growth defect of the bacteria, which would also be disadvantageous for their survival and host persistence [68]–[70]. As a consequence a complex feedback mechanism must be responsible for perfect adaptation. The molecular mechanism coupling transcription and translation with Yop export is currently under investigation.

## Material and Methods

### Ethics statements

Animal work was performed in strict accordance with the German regulations of the Society for Laboratory Animal Science (GV-SOLAS) and the European Health Law of the Federation of Laboratory Animal Science Associations (FELASA). The protocol was approved by the Niedersächsisches Landesamt für Verbraucherschutz und Lebensmittelsicherheit: animal licensing committee permission no. 33.9.42502-04-055/09.

### Cell culture, media and growth conditions

*E. coli* and *Yersinia* strains were routinely grown under aerobic conditions at 25°C or 37°C in LB (Luria Bertani) broth on solid or in liquid media if not indicated otherwise. The antibiotics used for bacterial selection were as follows: ampicillin 100 µg ml^−1^, chloramphenicol 30 µg ml^−1^, tetracyclin 10 µg ml^−1^, and kanamycin 50 µg ml^−1^.

### Strain and plasmid constructions

All DNA manipulations, polymerase chain reactions, restriction digestions, ligations and transformations were performed using standard genetic and molecular techniques [71]–[72]. Plasmid DNA was purified using the Qiagen Plasmid Mini or Midi Kits. Restriction and DNA-modifying enzymes were obtained from Roche, Fermentas, Promega or New England Biolabs. The oligonucleotides used for amplification by PCR, sequencing and primer extension were purchased from Metabion. PCR reactions were performed routinely in a 100 µl mix for 25 cycles using *Taq* polymerase or Phusion High-Fidelity DNA polymerase (New England Biolabs) according to the manufacturer's instructions. PCR products were purified with the QIAquick PCR purification kit (Qiagen) before and after digestion of the amplification product. Site-directed mutagenesis to delete or substitute nucleotides in the *yscW-lcrF* intergenic region of pBO1817 and pBO1818 was performed as described in the instruction manual of the QuikChange mutagenesis kit (Stratagene, LaJolla, USA) with plasmids harboring the *yscW-lcrF* wildtype sequence as template and the mutagenic primers listed in Table **S1**. Sequencing reactions were performed by GATC (Konstanz, Germany) or by the in-house facility.

Strains and plasmids used in this study are listed in Table **1** and primers for plasmid generation are listed in Table **S1**. The *ymoA* ^+^ fragment of *Y. pseudotuberculosis* of pAKH71 was generated by PCR using primers 1 and 2, digested with *Bam*HI and *Sal*I, and inserted into pACYC184. To construct pAKH77 a DNA fragment carrying the *ymoA* gene was amplified by PCR using the primer pair 79/80. The fragment was digested with *Eco*RI and *Xho*I and inserted into the corresponding sites of pASK-IBA5plus. A fragment carrying the *yscW-lcrF* intergenic region and the first 10 nt of the *lcrF* gene under control of the T7 promoter was amplified with primer pair 3/4. The resulting fragment was cloned into the *Sma*I site of pUC18 to generate plasmid pBO1817. Plasmid pBO1823 and pBO1855 were derived from pBO1817 by site-specific mutagenesis using the mutagenesis primer pairs 5/6 and 7/8, respectively. For toeprinting analysis pBO1818 was constructed by insertion of a PCR fragment amplified with primer pair 9/10 into the *Sma*I site of pUC18. Plasmids pBO1824 and pBO1855 were derived from pBO1818 by site-specific mutagenesis using the mutagenesis primer pairs 11/12 and 13/14, respectively. Primer 15 and 16 were used for amplification of the 5′-untranslated region of the *gnd* gene of *E. coli* from chromosomal DNA of MC4100 and the resulting fragment was cloned into the *Nhe*I/*Eco*RI sites of pBAD18-lacZ(481) to generate pED05. For construction of plasmids pED06–pED08 and pED12–pED13 harboring different mutations within the *yscW-lcrF* intergenic region upstream of the *lcrF-lacZ* fusion on pKB14, a two-step PCR was performed. The first PCR reaction was always performed with primer 17 and mutagenesis primer I, and the second PCR with primer 18 and mutagenesis primer II. The following listed mutagenesis primer I/II were used for plasmid: pED06 (19/20), pED07 (21/22), pED08 (23/24), pED12 (25/26), and pED13 (27/28). The two generated PCR fragments for each plasmid were used as templates, amplified with primer pair 17 and 18, and cloned into the *Nhe*I/*Eco*RI site of pBAD18-*lacZ*(481). Plasmids pED10 and pED11 contain *lcrF-lacZ* fusions with different portions of the *yscW* locus located upstream of the *lcrF* gene, starting from position +5 and +246 relative to the *yscW* start codon. For generation of the different fusion fragments, primer combination 25/8, and 26/8 and template plasmid pSF4 was used for PCR, and the resulting fragments were cloned into the *Nhe*I/*Eco*RI sites of pBAD18-*lacZ*(481). To analyze expression of the *yscW* gene, a PCR-derived fragment harboring the *yscW* regulatory region from position −310 to +281 relative to the transcriptional start site of *yscW* was amplified from chromosomal DNA of *Y. pseudotuberculosis* strain YPIII with primers 32 and 33 and cloned into the *Pst*I site of pGP20 to generate pKB10. To engineer an in frame deletion of *yscW* from nucleotide position +378 to +564 in the *yscW-lcrF-lacZ* fusion, first a two-step PCR was performed. In the first step, two fragments containing the region upstream and downstream of the *yscW* deletion was amplified from chromosomal DNA of YPIII using primer pair 32/73 and 74/37. Subsequently a third PCR was performed with primers 32 and 37 using the upstream and the downstream fragments as templates. The PCR product was digested with *Pst*I and ligated into the vector pGP20 generating pKB12. To construct pKB14, the *yscW-lcrF* intergenic region was amplified with primer pair 17 and 18 and cloned into the *Nhe*I/*Eco*RI sites in plasmid pBAD18-*lacZ*(481). To obtain equivalent plasmids (pKB13 and pKB18) in which hairpin II (Δ-44/-25) or hairpin I (Δ-111/-57) was deleted from the *yscW-lcrF* intergenic region, primer pairs 17/34 and 18/35 or 17/42 and 18/43 were used to synthesize overlapping fragments which were used for a third amplification reaction with primer pair 17 and 18. The resulting PCR fragments were also inserted into the *Nhe*I/*Eco*RI sites of pBAD18-*lacZ*(481). Plasmid pKB34 carrying a *yscW-lcrF*-*lacZ* fusion starting from position −310 was constructed by insertion of a PCR fragment amplified with primer pair 36/37 into the *Pst*I site of pTS02. Continuous deletions of the 5′-regulatory region were obtained by amplification using different forward primers 38–41 and reverse primer 37. The resulting fragments were ligated into the *Pst*I site of pTS02 to generate the *yscW-lcrF-lacZ* fusion plasmids pKB39–42. Plasmids pKB84 and pKB85 were constructed by amplification of the *yscW* upstream region either without (pKB84) or with the *yscW* 5′-UTR (pKB85) using primer pairs 65/66 or 65/67, respectively. The resulting PCR fragments were cloned into the *Kpn*I site of pFU68. To construct plasmid pKB90 harboring a deletion of the 5′-untranslated region (5′-UTR) of *yscW* from +13 to +241 relative to the transcriptional start site, a two-step PCR reaction was performed. Two fragments containing the region upstream and downstream of the *yscW* 5′-UTR were amplified from chromosomal DNA of YPIII using primer pairs 87/89 and 88/90. Subsequently a third PCR was performed with primers 87 and 88 using the upstream and the downstream fragments as templates. The product was digested with *Pst*I introduced into pTS02. To engineer a deletion of *yscW* from nucleotide position +271 to +651 two fragments containing the region upstream and downstream of the *yscW* deletion were amplified from chromosomal DNA of YPIII using primer pairs 75/77 and 76/78. Subsequently, a third PCR was performed with primers 77 and 78 using the upstream and the downstream fragments as templates. The product was digested with *Spe*I and *Sph*I and introduced into pDM4 resulting in pRS29. For construction of plasmids pRS41–46 harboring different mutations within the *yscW-lcrF* intergenic region upstream of the *lcrF* gene, a two-step PCR was performed and the resulting fragments were cloned into the *Sph*I/*Spe*I sites in pDM4. For the construction of pRS41, first two PCR reactions were performed with primers 46/50 and 47/51, and the two PCR fragments were used as templates to amplify the *yscW-lcrF* (AG-46/-45CC) mutant version with primer pair 44/45. Plasmids pRS42–44 were constructed by insertion of *Spe*I/*Sph*I fragments amplified with primers 44/45 from different PCR fragments used as templates. For the production of the template fragments the two primer pairs 44/52 and 45/53, 44/54 and 45/55 as well as 44/56 and 45/57 were used for the synthesis of the GUU-30/-28AAA, UU-28/27CC and AUA-36/-34CCC fragments. To engineer plasmid pRS45, (Δloop2 −44/−25) both template fragments were obtained by amplification with primers 48/49 and 64/49. After annealing of the template fragments, the *yscW-lcrF* fragment harboring the Δloop2 −44/−25 mutation was amplified with primer pair 63/64 and cloned into the *Spe*I/*Sph*I sites of pDM4. For the construction of pRS46, first two PCR reactions were performed with primers 48/43 and 49/42, and the two PCR fragments were used as templates to amplify the *yscW-lcrF* (AG-46/-45CC) mutant version with primer pair 48/49. The *yadA-lacZ*translational fusion encoded by pSF1 was constructed by insertion of a *yadA* promoter fragment amplified with the primer pair 91/92 into the *Pst*I site of pGP20. The *lcrF-lacZ* and *yscW-lcrF-lacZ* fusion plasmids pSF3 and pSF4 were constructed by insertion of PCR fragments amplified from *Y. pseudotuberculosis* YPIII genomic DNA with primer pairs 37/58 and 37/32 into the *Pst*I site of pGP20.

**Table 1:** Bacterial strains and plasmids.

Strains, Plasmids	Description	Source and reference
**Bacterial strains**		
*E. coli* K-12		
CC118 λpir	F^−^ Δ(*ara-leu*)7697 Δ(*lacZ*)74 Δ(*phoA*)20 *araD139*	[82]
	*galE galK thi rpsE rpoB arfE* ^am^ *recA1*, λpir	
BL21 λDE3	F^−^ *ompT gal dcm lon hsdSB*(r_B_^−^ m_B_^−^) *gal* λDE3	[83]
KB1	BL21 λDE3 (Δ*stpA*)	This work
KB3	KB1 (Δ*hns*)	This work
KB4	KB3 (Δ*hha*)	This work
*Y. pseudotuberculosis*		
IP32953	pYV, wildtype	[84]
YPIP01	IP32953, *yscW-lcrF* (UU-28/-27CC)	This work
YPIP02	IP32953, *yscW-lcrF* (GUU-30/-28AAA)	This work
YPIII	pIB1, wildtype	[85]
YP50	YPIII, Δ*ymoA,* Kn^R^	This work
YP66	YPIII, Δ*lcrF,* Ap^R^	This work
YP82	YPIII, *yscW-lcrF* (AG-46/-45CC), Kn^R^	This work
YP83	YPIII, *yscW-lcrF* (UU-28/-27CC), Kn^R^	This work
YP84	YPIII, *yscW-lcrF* (GUU-30/-28AAA), Kn^R^	This work
YP85	YPIII, *yscW-lcrF* (AUA-36/-34CCC), Kn^R^	This work
YP86	YPIII, *yscW-lcrF* (Δloop1 −111/−57), Kn^R^	This work
YP90	YPIII, *yscW-lcrF* (UU-28/-27CC)	This work
YP94	YPIII, *yscW-lcrF* (Δloop2 −44/−25)	This work
YP95	YPIII, *yscW-lcrF* (GUU-30/-28AAA)	This work
YP96	YPIII, *yscW*(Δ271–651)*-lcrF*	This work
**Plasmids**		
pACYC177	cloning vector, p15A, Ap^R^, Kan^R^	[86]
pACYC184	cloning vector, p15A, Cm^R^, Tet^R^	[86]
pAKH11	pET28, *hns* ^+^, Kn^R^	[76]
pAKH71	pACYC184, *ymoA* ^+^, Cm^R^	This work
pAKH74	pACYC184, *hns*+, Cm^R^	[74]
pAKH77	pASK-IBA5plus, *ymoA* ^+^, Ap^R^	This work
pASK-IBA5plus	*tetR*, *tet* p/o, N-terminal-Strep-Taq-fusion, Ap^R^	IBA GmbH
pBAD18-lacZ(481)	pBAD18, P*_BAD_*::*lacZ*, Ap^R^	[55]
pBO1817	pUC18, T7-*lcrF*′ (−123 to 10), Ap^R^	This work
pBO1818	pUC18, T7-*lcrF*′ (−123 to 63), Ap^R^	This work
pBO1823	pUC18, T7-*lcrF*′ (−123 to 10, UU-28/-27CC), Ap^R^	This work
pBO1824	pUC18, T7-*lcrF*′ (−123 to 63, GUU-30/-28AAA), Ap^R^	This work
pBO1833	pUC18, T7-*lcrF*′ (−123 to 63, UU-28/-27CC), Ap^R^	This work
pBO1855	pUC18, T7-*lcrF*′ (−123 to 10, GUU-30/-28AAA), Ap^R^	This work
pCP20	FLP recombinase expression vector, Ap^R^, Cm^R^	[75]
pDM4	R6K derivative, *sacB*, Cm^R^	Debra Milton
pED05	pBAD18-lacZ(481), P_BAD_::*gnd*-*lacZ,* Ap^R^	This work
pED06	pKB14, P*_BAD_*::*lcrF-lacZ* (AG-46/-45CC)^a^, Ap^R^	This work
pED07	pKB14, P*_BAD_*::*lcrF-lacZ* (GUU-30/-28AAA)^a^, Ap^R^	This work
pED08	pKB14, P*_BAD_*::*lcrF-lacZ* (UU-28/-27CC)^a^, Ap^R^	This work
pED10	pBAD18, *yscW-lcrF-lacZ* (+269^b^; +74^a^), Ap^R^	This work
pED11	pBAD18, *yscW-lcrF-lacZ* (+510^b^; +74^a^), Ap^R^	This work
pED13	pKB14, P*_BAD_*::*lcrF-lacZ* (AUA-36/-34CCC)^a^, Ap^R^	This work
pET28a	T7 overexpression vector, Kn^R^	Novagen
pFU68	pSC101*, *lacZ*, Ap^R^	[87]
pGP20	pSC101, promoterless *lacZ* gene, Tet^R^	P. Gerlach
pKB10	pGP20, *yscW-lacZ* (−310 to +281)^b^, Ap^R^	This work
pKB12	pGP20, *yscW*(Δ378–564)*-lcrF-lacZ*, Tet^R^	This work
pKB13	pBAD18, *lcrF-lacZ* (Δloop2 −44/−25)^a^, Ap^R^	This work
pKB14	pBAD18-lacZ(481), P*_BAD_*::*lcrF-lacZ* (−124;+74)^b^, Ap^R^	This work
pKB18	pKB14, P*_BAD_*::*lcrF-lacZ* (Δloop1 −111/−57)^a^, Ap^R^	This work
pKB34	pTS02, *yscW-lcrF-lacZ* (−310)^b^, Ap^R^	This work
pKB39	pTS02, *yscW-lcrF-lacZ* (−211)^b^, Ap^R^	This work
pKB40	pTS02, *yscW-lcrF-lacZ* (−103)^b^, Ap^R^	This work
pKB41	pTS02, *yscW-lcrF-lacZ* (−2)^b^, Ap^R^	This work
pKB42	pTS02, *yscW-lcrF-lacZ* (+94)^b^, Ap^R^	This work
pKB84	pFU68, *yscW*-*lacZ* (−1016 to −265)^b^, Ap^R^	This work
pKB85	pFU68, *yscW*-*lacZ* (−1016 to −15)^b^, Ap^R^	This work
pKB90	pTS02, *yscW-lcrF-lacZ* (−310, Δ13–241), Ap^R^	This work
pKD4	kanamycin cassette template, Kn^R^	[75]
pKD46	recombination vector, λ RED recombinase, Ap^R^	[75]
pKOBEG-*sacB*	recombination vector, *sacB* ^+^, Cm^R^	[73]
pRS29	pDM4, *yscW*(Δ271–651), Cm^R^	This work
pRS41	pDM4, *lcrF* (AG-46/-45CC)^a^, Cm^R^	This work
pRS42	pDM4, *lcrF* (GUU-30/-28AAA)^a^, Cm^R^	This work
pRS43	pDM4, *lcrF* (UU-28/-27CC)^a^, Cm^R^	This work
pRS44	pDM4, *lcrF* (AUA-36/-34CCC)^a^, Cm^R^	This work
pRS45	pDM4, *lcrF* (Δloop2 −44/−25)^a^, Cm^R^	This work
pRS46	pDM4, *lcrF* (Δloop1 −111/−57)^a^, Cm^R^	This work
pSF1	pGP20, *yadA-lacZ*, Tet^R^	This work
pSF3	pGP20, *lcrF-lacZ* (+257^b^; +74^a^), Tet^R^	This work
pSF4	pGP20, *yscW-lcrF-lacZ* (−310^b^;+74^a^), Tet^R^	This work
pTS02	pGP20, Ap^R^	Tatjana Stolz
pWO34	pSC101*, *yopE-luxCDABE*, Cm^R^	[87]

a The number indicates the nucleotide of *lcrF*, relative to the *lcrF* start codon.

b The number indicates the nucleotide of *yscW*, relative to the *yscW* transcriptional start site.

### Construction of the *E. coli* and *Y. pseudotuberculosis* deletion mutants

*Y. pseudotuberculosis* strain YP50 was constructed by insertion of a kanamycin cassette into the locus of wildtype strain YPIII using the RED recombinase system as described [73]. First, the kanamycin resistance gene was amplified using the kan*_ymoA_* primers (Table **S2**) and plasmid pACYC177 as template. Next, the *Yersinia* genomic DNA was used as a template to amplify 500-bp regions flanking the target gene. The upstream fragment was amplified with a primer pair of which the reverse primer contained additional 20 nt at the 5′-end which were homologous to the start of the kanamycin resistance gene. The downstream fragment was amplified with a primer pair of which the forward primer contained additional 20 nt at the 3′-end which were homologous to the end of the kanamycin resistance gene (for primer see Table **S2**). In the next step, a PCR reaction was performed with the forward primer and the reverse primer using the upstream and downstream PCR products of the target gene and the *kan* gene fragment as templates. The PCR fragment was transformed into *Y. pseudotuberculosis* YPIII pKOBEG-*sacB* and chromosomal integration of the fragments was selected by plating on LB supplemented with kanamycin. Mutants cured of pKOBEG-*sacB* were proven by PCR and DNA sequencing.

For the construction of the *lcrF* knock-out mutant strain YP66, a *lcrF*::Amp^R^ PCR fragment was generated using an Amp^R^-resistance plasmid as a template with primers composed of 55 nucleotides which are homologous to the up- or downstream region of the *lcrF* gene followed by 20 nucleotides homologous to the 5′- or 3′-end of the ampicillin resistance gene (for primer see Table **S2**). The resulting PCR fragment was integrated into the *lcrF* locus of *Y. pseudotuberculosis* YPIII on pYV by the RED recombinase system (Derbise *et al*., 2003). Selection of the mutant strain was performed as described [74]. One strain, YP66, harboring the *lcrF*::Amp^R^ mutation in the *lcrF* locus, as proven by PCR and DNA sequencing, was used for further studies.

All mutant strains with deletions or nucleotide substitutions in the *yscW-lcrF* intergenic region (YP82–86, YP90, YP95, YP96, YPIP01 and YPIP02) were constructed by homologous recombination using suicide plasmids pRS41–46 and pRS29. Plasmids were mated from *E. coli* S17-1 λpir (*tra* ^+^) into *Y. pseudotuberculosis* YPIII or IP32953 and transconjugants were selected on *Yersinia* selective agar (Oxoid) supplemented with chloramphenicol. The recombination of the plasmid into the *Yersinia* virulence plasmid pYV yielded a merodiploid strain, including a wildtype and the mutant copy of *yscW-lcrF*. Subsequently, the resulting strain was plated on 10% sucrose and fast growing, large colonies were selected. Because sucrose induces the expression of the *sacB* gene on the integrated plasmids and leads to the production of a toxic substance that prevents growth, a spontaneous second recombination process resulting in the excision of the integrated plasmid is advantageous. 50 selected fast-growing strains were screened for chloramphenicol sensitivity to prove the loss of the integrated plasmid. One strain (Table **1**), harboring the desired *yscW-lcrF* mutation, as proven by PCR and DNA sequencing, was taken for further analysis.

The *E. coli* mutant strains were constructed with the RED recombinase system as described previously [75]. *E. coli* strain KB1 was constructed by introducing a *stpA* deletion into strain BL21lDE3 and used to generate KB3 (BL21λDE3 *stpA* ^−^ *hns* ^−^). Subsequently, KB3 was used to construct KB4 (BL21λDE3 *stpA* ^−^ *hns* ^−^ *hha* ^−^). First, a kanamycin cassette was amplified by PCR with primers homologous to the resistance gene encoded on pKD4 followed by homologous sequences of adjacent regions of the target gene (for primer see Table **S2**). The PCR fragment was transformed into *E. coli* BL21 pKD46. Chromosomal integration of the fragment was selected by plating on LB supplemented with kanamycin. Subsequently, mutant derivatives were cured of the temperature-sensitive plasmid pKD46 by cultivation at 37°C. To remove the resistance gene at its FLP recognition sites the mutants were transformed with the helper plasmid pCP20 encoding the FLP recombinase. For thermal induction of FLP synthesis and subsequent removal of the temperature-sensitive plasmid pCP20, mutants were incubated at 37°C.

### RNA isolation and Northern detection

Overnight cultures were diluted 1/50 in fresh medium and grown to stationary phase (OD_600_ of 3). 2.5 ml culture were withdrawn, mixed with 0.2 volume of stop solution (5% water-saturated phenol, 95% ethanol) and snap-frozen in liquid nitrogen. After thawing on ice, bacteria were pelleted by centrifugation (2 min, 14.000 rpm, 4°C), and RNA was isolated using the SV total RNA purification kit (Promega) as described by the manufacturer. RNA concentration and quality were determined by measurement of A_260_ and A_280_.

Total cellular RNA (20 µg) was separated on MOPS agarose gels (1.2%), transferred by Vacuum Blotting for 1.5 h onto positively charged membranes (Whatman) in 10× SSC using a semi-dry blotting system and UV cross-linked. Prehybridization, hybridization to DIG-labelled probes and membrane washing were conducted using the DIG luminescent Detection kit (Roche) according to the manufacturers instructions. The *yscW*-*lcrF* transcript was detected with a DIG-labelled PCR fragment (DIG-PCR nucleotide mix, Roche) with primer pair 59 and 60 (Table **S1**).

### Expression and purification of the *Y. pseudotuberculosis* YmoA and H-NS protein

KB4 transformed with pAKH77 or pAKH11 was grown at 37°C in LB broth to an A_600_ of 0.6. Anhydrotetracycline was added (0.2 µg/ml) to induce the expression of YmoA-Strep-Tag or 2 mM IPTG was used to induce H-NS-His_6_ expression. For purification of the YmoA-H-NS heterodimer KB4 transformed with pAKH74 and pAKH77 was used for overexpression of the YmoA in the presence of the *Yersinia* H-NS protein. The cells were grown for an additional 3 h before being harvested. The purification procedure for the Strep-tagged YmoA protein was performed according to the manufacturers instructions (IBA GmbH, Germany). H-NS purification was performed as described [76]. The purity of the YmoA and the H-NS protein was estimated to be >95%.

### Gel retardation assays

For DNA-binding studies the purified YmoA and H-NS proteins were dialysed against the DNA-binding buffer (10 mM Tris-HCl pH 7.5; 3 mM DTT; 7,5% glycerol; 100 mM KCl; 100 mM MgCl_2_). The *yscW* fragment (−2 to +272) for DNA band shift analysis was obtained by PCR using primer pair 68/69 with chromosomal DNA of *Y. pseudotuberculosis* YPIII and the *csiD1* and *csiD2* control fragments were amplified by PCR from chromosomal DNA of *E. coli* strain MC4100 with primer pairs 71/72 and 70/71 (see Table **S1**). The DNA fragments and increasing concentrations of purified YmoA or H-NS were incubated for 30 min in DNA-binding buffer at room temperature and immediately loaded on 4% polyacrylamide gels.

### *In vitro* transcription and structure probing of the *lcrF* RNA

The *lcrF* RNA for structural probing was obtained by run-off transcription with T7 RNA polymerase from plasmids pBO1817, pBO1823 and pBO1855 linearized with *Mls*I. The RNAs were radioactively 5′-end labelled according to Brantl & Wagner [77]. Partial digestion of the RNAs was performed using the ribonucleases T1 and V (Ambion, USA) as described previously [43]. RNA corresponding to about 30.000 cpm was mixed with 1 µl of 5× TMN buffer (100 mM tris acetate pH7.5, 10 mM MgCl_2_, 500 mM NaCl), 0.5 µg tRNA (Invitrogen, Germany), and destilled water to a total volume of 4 µl. The samples were incubated at 25°C or 37°C for 5 min before 1 µl of RNAse T1 (0.001 U/µl), RNase V (0.0002 U/µl) or RNAse-free water were added. After 5 min of digestion at the appropriate temperature, the reaction was stopped by addition of 5 µl formamide stop solution. The samples were denatured at 95°C, and separated on a denaturing 8% poyacrylamide/urea gel. The alkaline ladders were generated with 60.000 counts of *lcrF* mRNA as described previously [77].

### Toeprinting analysis

RNAs for primer extension inhibition experiments (toeprinting analysis) were synthesized *in vitro* by runoff transcription with T7 RNA polymerase from linearized plasmids pBO1818, pBO1824, and pBO1833. Toeprinting experiments were performed with 30S ribosomal subunits, *lcrF* mRNA and tRNA^fMet^ mainly as described previously [43]. The 5′-[P^32^]-labelled *lcrF*-specific primer 61 (2 pmol) was used for reverse transcriptase reaction. About 1 pmol of the *lcrF* mRNA was annealed to the radioactive primer and incubated for 20–30 min at 25°C and 37°C in a 20 µl reaction mix with 16 pmol of uncharged tRNA^fMet^ (Sigma-Aldrich, USA) in the presence or absence of 6 pmol of the 30S ribosomal subunits isolated as described [78]. To initiate the primer extension reaction, 2 µl VD-Mg^2+^ buffer (0.05 M Tris-HCl pH 7.4, 0.3 M NH_4_Cl, 30 mM β-mercaptoethanol, 0.05 M MgO acetate) containing 80 U of the MMLV reverse transcriptase (USB, USA), dNTPs, and BSA was added and incubated for 10 min at 25°C. cDNA synthesis was stopped by the addition of 20 µl formamide stop solution. In parallel, sequencing reactions using the same *lcrF* specific primer was performed with the Thermo Sequenase cycle sequencing Kit (USB, USA) according to the manufacturer's instructions. All samples were denatured at 95°C for 5 min, and separated on a denaturing 8% polyacrylamide/urea gel.

### Primer extension analysis to identify the *yscW-lcrF* transcriptional start site

Primer extension analysis was performed to determine the transcriptional start site of the *yscW-lcrF* mRNA from strain YPIII. At an OD_600_ of 2.0 (early stationary phase), total RNA was extracted of the samples using the SV total RNA purification kit (Promega) as described by the manufacturer. Annealing was performed with 20 µg extracted RNA and the 5′-Dig-labelled oligonucleotide 62 for *yscW* in 20 µl of 1× First Strand Buffer (Invitrogen) by slow cooling of the sample (0.01°C/sec). 8 mM dNTPs and 5× FS Buffer (Invitrogen) with 200 U Superscript II reverse transcriptase (Invitrogen) was added to the reaction mix and incubated for 1 h at 42°C. The size of the Dig-labelled reaction products was determined on a denaturing 6% DNA sequencing gel by a detection procedure as described [79].

### Analysis of the reporter gene expression

The activity of the β-galactosidase activity of the *lacZ* and the *phoA* fusion constructs was measured in permeabilized cells as described previously [71], [80]. The activities were calculated as follows: β-galactosidase activity OD_420_ · 6,75 · OD_600_^−1^ ·Δt (min)^−1^ · Vol (ml)^−1^; alkaline phosphatase activity OD_420_ · 6,46 · OD_578_^−1^ · Δt (min)^−1^ · Vol (ml)^−1^. Reporter fusions emitting bioluminescence were measured in non-permeabilized cells with a Varioskan Flash (Thermo Scientific) using the SkanIt software (Thermo Scientific) for 1 s per time point. The data are given as relative light units (RLU/OD_600_) from three independent cultures performed in duplicate. The level of statistical significance for differences in reporter gene expression was determined by the Student's t test.

### Gel electrophoresis, preparation of cell extracts and Western blotting

For immunological detection of the LcrF and YadA proteins, *Y. pseudotuberculosis* cultures were grown under specific environmental conditions as described. Cell extracts of equal amounts of the bacteria were prepared and separated on a 15% (LcrF) or 10% (YadA) SDS-PAGE [72]. Subsequently the samples were transferred onto an Immobilon-P membrane (Millipore) and probed with polyclonal antibodies directed against YadA or LcrF (generous gift of Greg Plano) as described [74]. The cell extracts used for Western blotting were also separated by SDS-PAGE and stained with Coomassie blue to ensure that the protein concentrations in the different cell extracts are comparable; about 10 mg protein was applied of each sample.

### Yop secretion assay

The Yop secretion assay was performed as described previously [69]. Bacteria were grown overnight at 25°C in LB medium, diluted 1∶50 in fresh LB medium and grown at 25°C until the culture reached an OD_600_ of about 0.4–0.5. Subsequently, the cultures were shifted to 37°C for 3–4 h in the absence or presence of 20 mM Mg^2+^ and 20 mM Naoxalate, a Ca^2+^ chelator. Proteins in the medium supernatant were harvested, filtered and precipitated with TCA. Precipitated proteins were resuspended in equal amounts of sample buffer, separated on 15% SDS polyacrylamide gels and visualized by Coomassie brilliant blue staining.

### Mouse infections

In order to assess the impact of the RNA thermometer on *Y. pseudotuberculosis* virulence, groups of 7-week-old female BALB/c mice were orally infected with 5·10^8^ bacteria using a ball-tipped feeding needle. To prepare the inocula, the *Y. pseudotuberculosis* wildtype strain YPIII and isogenic mutant strains were cultured overnight in LB at 25°C. The bacteria were harvested by centrifugation, washed and resuspended to the appropriate concentration in PBS. Three days after infection of the mice the colony forming units (CFU) per gram tissue were determined in the Peyer's patches, mesenterial lymph nodes, liver and spleen. Isolated Peyer's patches were rinsed with sterile PBS and incubated with 100 µg/ml gentamicin in PBS in order to kill the bacteria on the luminal surface. After 30 min, gentamicin was removed by extensive washing with PBS for three times. Subsequently, all organs were weighted, homogenized in PBS, and plated in three independent serial dilutions on *Yersinia* selective agar (Oxoid, Germany). For the survival assays, groups (*n* = 10) of 7-week-old BALB/c mice were orally infected with a lethal dose of 2·10^9^ bacteria (*Y. pseudotuberculosis* strains YPIII and mutant strains cultured overnight in LB at 25°C). The infected mice were monitored for 14 days every day to determine the survival rate.

## Supporting Information

Figure S1 **Identification of regulatory sequences important for** ***yscW-lcrF*** **expression.** (**A**) Strains YPIII and YP50 (Δ*ymoA*) expressing *yscW-lcrF-lacZ* fusions with different 5′-deletions of the *yscW* regulatory region were grown overnight in LB medium at 25°C. β-Galactosidase activity from overnight cultures was determined and is given in µmol min^−1^ mg^−1^ for comparison. The data represent the average ± SD from at least three different experiments each done in duplicate. Data were analyzed by the Student's t test. Stars indicate the results that differed significantly between the different 5′-deletion constructs in the wildtype or the *ymoA* mutant strain with ** (P<0.01), and *** (P<0.001). (**B**) Influence of YmoA on the 5′ UTR of *yscW*. Strains YPIII and YP50 (Δ*ymoA*) expressing *yscW* transcriptional fusions either with the promoter region and the 5′ UTR (pKB85) or only the promoter region (pKB84) were grown overnight in LB medium at 25°C. β-Galactosidase activity from overnight cultures was determined and is given in µmol min^−1^ mg^−1^ for comparison. The data represent the average ± SD from at least three different experiments each done in duplicate. Data were analyzed by the Student's t test. Stars indicate the results that differed significantly between the different 5′-deletion constructs in the wildtype or the *ymoA* mutant strain with *** (P<0.001).
(TIF)

Figure S2 **Gel retardation experiments using purified YmoA, H-NS and YmoA copurified with H-NS.** Individual DNA fragments comprising the *yscW* upstream region and the different portion of the *E. coli csiD* gene as negative control (*csiD1* and *csiD2*) were incubated without protein or with increasing amounts of purified *Y. pseudotuberculosis* YmoA (**A**), H-NS (**B**) or YmoA purified in the presence of H-NS (YmoA_H-NS_) protein (**C**). The samples were separated on a 4% polyacrylamide gel, a molecular weight standard (M: 100 bp ladder) was loaded, and the corresponding molecular weights are indicated on the left. The positions of the DNA fragments and the higher molecular weight protein-DNA complexes are indicated. (**D**) Strains YPIII and YP50 (Δ*ymoA*) expressing a *yscW-lcrF-lacZ* (pKB34) or a *yscW*(Δ13–241)-*lcrF-lacZ* fusion (pKB90) with a deletion of the 5′-UTR of the *yscW* regulatory region were grown overnight in LB medium at 25°C and 37°C. β-galactosidase activity from overnight cultures was determined and is given in µmol min^−1^ mg^−1^ for comparison. The data represent the average ± SD from at least three different experiments each done in duplicate. Data were analyzed by the Student's t test. Stars indicate the results that differed significantly between the wildtype or the *ymoA* mutant strain with *** (P<0.001).
(TIF)

Figure S3 **The intergenic region of the** ***yscW-lcrF*** **operon is implicated in the temperature control of LcrF production.** YPIII harboring the different P*_BAD_*::*yscW-lcrF-lacZ* reporter plasmids (pED10, pED11 and pKB14) or the P*_BAD_*::*gnd-lacZ* control plasmid (pED05) were grown overnight in LB medium at 25°C or 37°C in the presence of 0.05% arabinose. β-Galactosidase activity from overnight cultures was determined and is given in µmol min^−1^ mg^−1^ for comparison. The data represent the average ± SD from at least three experiments each done in duplicate. Stars indicate the reporter activity that differed significantly between 25°C and 37°C with *** (P<0.001).
(TIF)

Figure S4 **Influence of** ***yscW*** **on LcrF production.** (**A**) YPIII harboring the different *yscW-lcrF-lacZ* or the *yscW*(Δ378–564)*-lcrF-lacZ* reporter plasmids pSF4 and pKB12 were grown overnight in LB medium at 25°C or 37°C. β-Galactosidase activity from overnight cultures was determined and is given in µmol min^−1^ mg^−1^ for comparison. The data represent the average ± SD from at least three experiments each done in duplicate. (**B**) YPIII and YP96 harboring a *yscW*(Δ271–651) were grown overnight in LB medium at 25°C or 37°C. Whole-cell extracts from overnight cultures were prepared and analysed by Western blotting with a polyclonal antibody directed against LcrF. A molecular weight marker is loaded on the left. A higher molecular weight protein (c) that reacted with the polyclonal antiserum was used as loading control.
(TIF)

Figure S5 **Influence of the** ***lcrF*** **RNA thermometer on tissue colonization by** ***Y. pseudotuberculosis*** **.** Strains YPIII (wildtype), the ‘closed’ *yscW-lcrF* variant YP90, and YP66 (Δ*lcr*F) were infected intragastrically (5·10^8^ CFU/mice) into BALB/c mice. After three days of infection, mice were sacrificed and the number of bacteria in homogenized host tissues and organs was determined by plating. Solid lines indicate the means. The statistical significances between the wildtype and the repressed and derepressed *lcrF* RNA thermometer variants were determined by the Student's t test. P-values: ***: <0.001.
(TIF)

Figure S6 **Influence of the** ***lcrF*** **RNA thermometer on tissue colonization by** ***Y. pseudotuberculosis*** **.** Strains IP32953 (wildtype), the ‘closed’ and open *yscW-lcrF* variant YPIP01 and YPIP02 were infected intragastrically (5·10^8^ CFU/mice) into BALB/c mice. After three days of infection, mice were sacrificed and the number of bacteria in homogenized host tissues and organs was determined by plating. Solid lines indicate the means. The statistical significances between the wildtype and the repressed and derepressed *lcrF* RNA thermometer variants were determined by the Student's t test. P-values: ***: <0.001.
(TIF)

Figure S7 **Multiple DNA sequence alignment of the** ***yscW-lcrF*** **operon of pathogenic** ***Yersinia*** **species.** The nucleotides identical in the sequences are indicated with dots. The alignment was created using ClustalW. The coding sequence of *yscW* and *lcrF* are given in green, the intergenic region (RNA thermometer sequence) is given in blue. The promoter regions are bold and underlined, the transcriptional and translational start sites as well as the stop codons are indicated in bold. *Y. pstb: Y. pseudotuberculosis*; *Y. pest*: *Y. pestis* and *Y. ent*: *Y. enterocolitica.*
(DOC)

Table S1 **Oligonucleotides used in this study.** The corresponding restriction sites are underlined, the T7 promoter region is given in italic, and introduced basepair substitutions are indicated in bold, the deletion of nucleotides is indicated by a short line, the sequence of kan resistance cassette is given in bold and underlined.
(DOC)

Table S2 **Primers used for the generation of deletion mutants.** ^a^ The *Y. pseudotuberculosis* mutants were constructed by adding a kanamycin resistance cassette (Kan). Underlined bases correspond to the homologous nucleotides of the resistance gene. Rev: reverse primer; for: forward primer.
(DOC)
