# Supplementary figures and images for: Concerted Actions of a Thermo-labile Regulator and a Unique Intergenic RNA Thermosensor Control Yersinia Virulence
Source: PLoS Pathog. 2012 Feb 16;8(2):e1002518. doi: 10.1371/journal.ppat.1002518 (PMC3280987; doi:10.1371/journal.ppat.1002518)

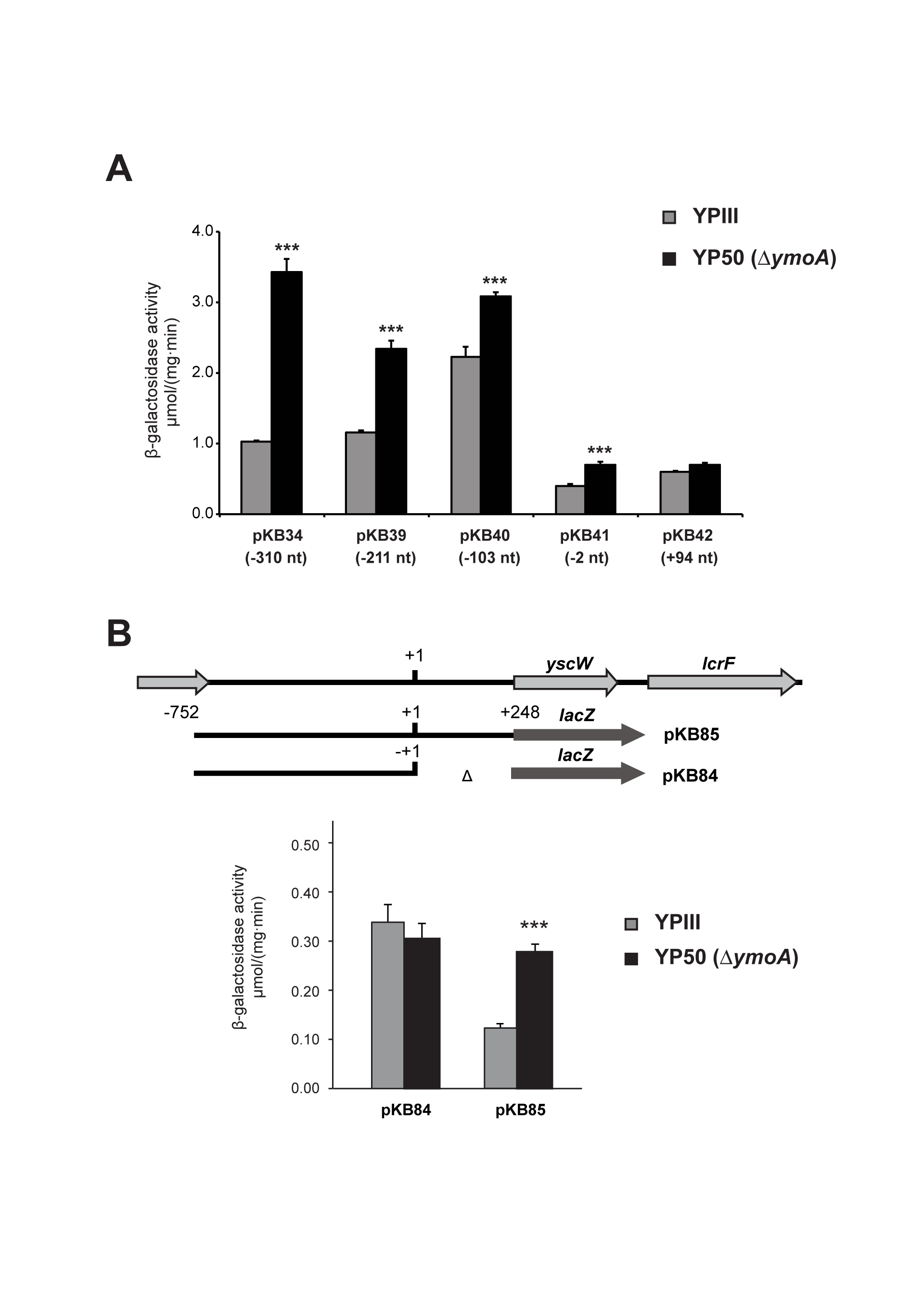

Supplement: Figure S1 — Identification of regulatory sequences important for yscW-lcrF expression. (A) Strains YPIII and YP50 (ΔymoA) expressing yscW-lcrF-lacZ fusions with different 5′-deletions of the yscW regulatory region were grown overnight in LB medium at 25°C. β-Galactosidase activity from overnight cultures was determined and is given in µmol min−1 mg−1 for comparison. The data represent the average ± SD from at least three different experiments each done in duplicate. Data were analyzed by the Student's t test. Stars indicate the results that differed significantly between the different 5′-deletion constructs in the wildtype or the ymoA mutant strain with ** (P<0.01), and *** (P<0.001). (B) Influence of YmoA on the 5′ UTR of yscW. Strains YPIII and YP50 (ΔymoA) expressing yscW transcriptional fusions either with the promoter region and the 5′ UTR (pKB85) or only the promoter region (pKB84) were grown overnight in LB medium at 25°C. β-Galactosidase activity from overnight cultures was determined and is given in µmol min−1 mg−1 for comparison. The data represent the average ± SD from at least three different experiments each done in duplicate. Data were analyzed by the Student's t test. Stars indicate the results that differed significantly between the different 5′-deletion constructs in the wildtype or the ymoA mutant strain with *** (P<0.001). (TIF) [file ppat.1002518.s001.tif]

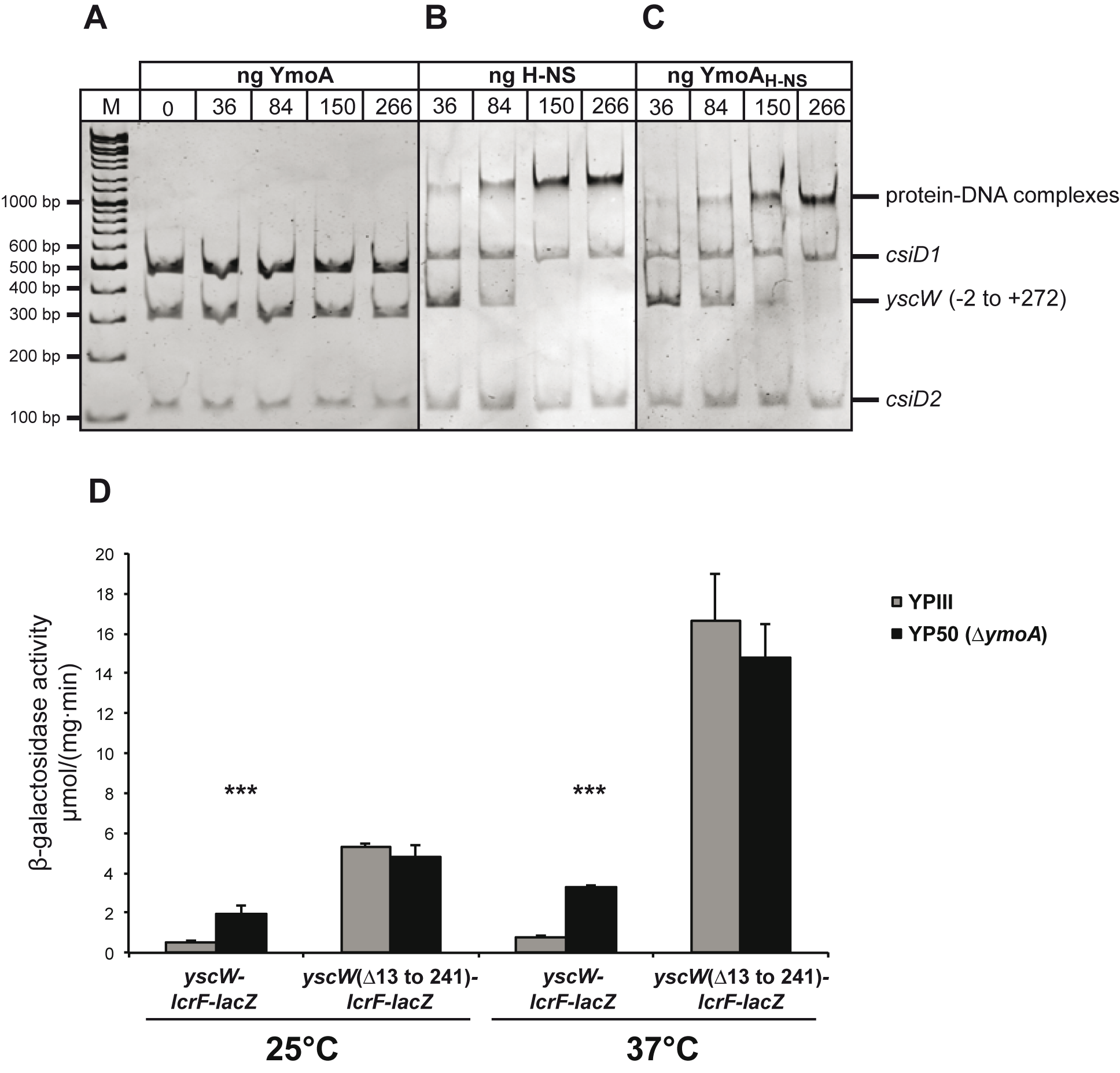

Supplement: Figure S2 — Gel retardation experiments using purified YmoA, H-NS and YmoA copurified with H-NS. Individual DNA fragments comprising the yscW upstream region and the different portion of the E. coli csiD gene as negative control (csiD1 and csiD2) were incubated without protein or with increasing amounts of purified Y. pseudotuberculosis YmoA (A), H-NS (B) or YmoA purified in the presence of H-NS (YmoAH-NS) protein (C). The samples were separated on a 4% polyacrylamide gel, a molecular weight standard (M: 100 bp ladder) was loaded, and the corresponding molecular weights are indicated on the left. The positions of the DNA fragments and the higher molecular weight protein-DNA complexes are indicated. (D) Strains YPIII and YP50 (ΔymoA) expressing a yscW-lcrF-lacZ (pKB34) or a yscW(Δ13–241)-lcrF-lacZ fusion (pKB90) with a deletion of the 5′-UTR of the yscW regulatory region were grown overnight in LB medium at 25°C and 37°C. β-galactosidase activity from overnight cultures was determined and is given in µmol min−1 mg−1 for comparison. The data represent the average ± SD from at least three different experiments each done in duplicate. Data were analyzed by the Student's t test. Stars indicate the results that differed significantly between the wildtype or the ymoA mutant strain with *** (P<0.001). (TIF) [file ppat.1002518.s002.tif]

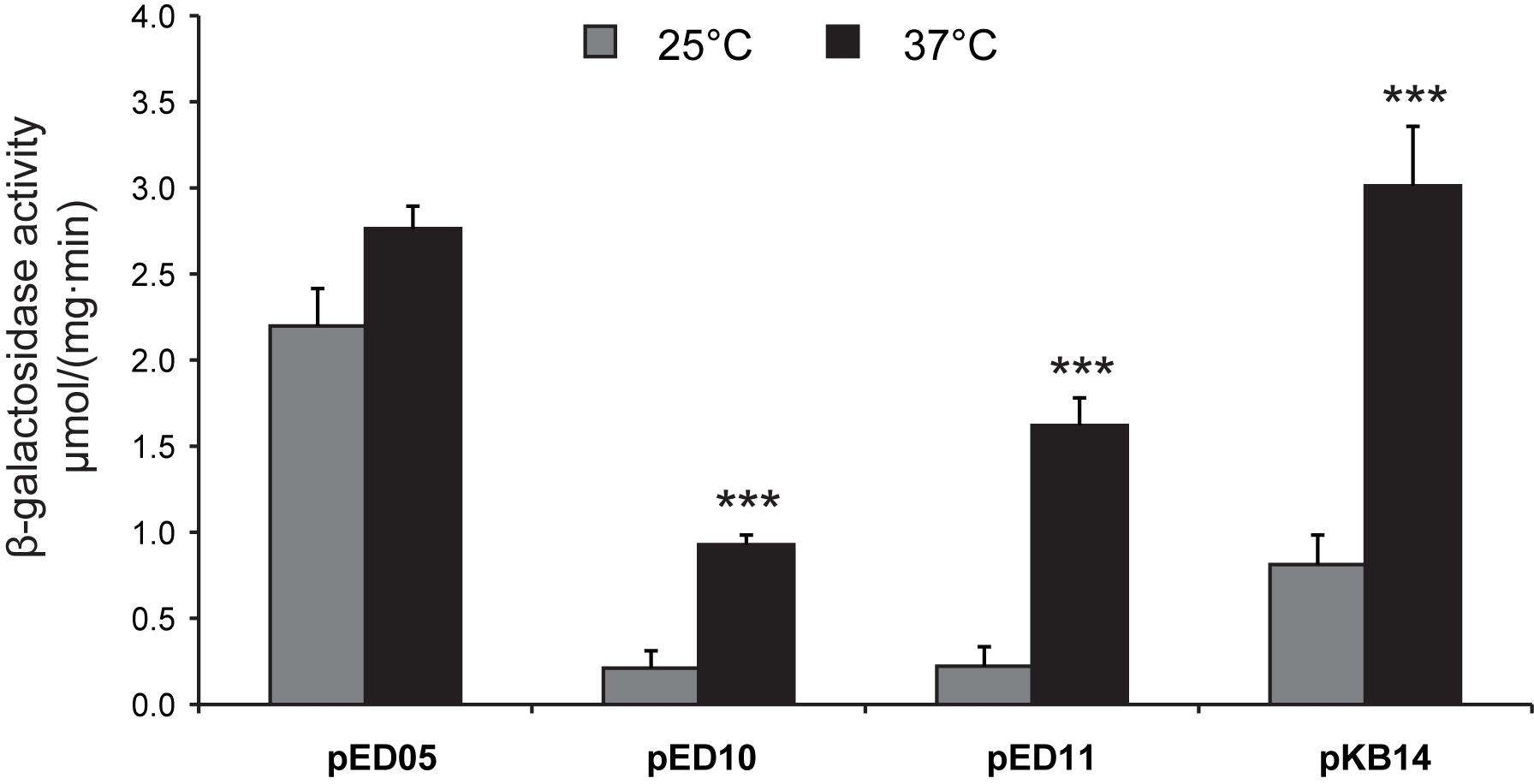

Supplement: Figure S3 — The intergenic region of the yscW-lcrF operon is implicated in the temperature control of LcrF production. YPIII harboring the different PBAD::yscW-lcrF-lacZ reporter plasmids (pED10, pED11 and pKB14) or the PBAD::gnd-lacZ control plasmid (pED05) were grown overnight in LB medium at 25°C or 37°C in the presence of 0.05% arabinose. β-Galactosidase activity from overnight cultures was determined and is given in µmol min−1 mg−1 for comparison. The data represent the average ± SD from at least three experiments each done in duplicate. Stars indicate the reporter activity that differed significantly between 25°C and 37°C with *** (P<0.001). (TIF) [file ppat.1002518.s003.tif]

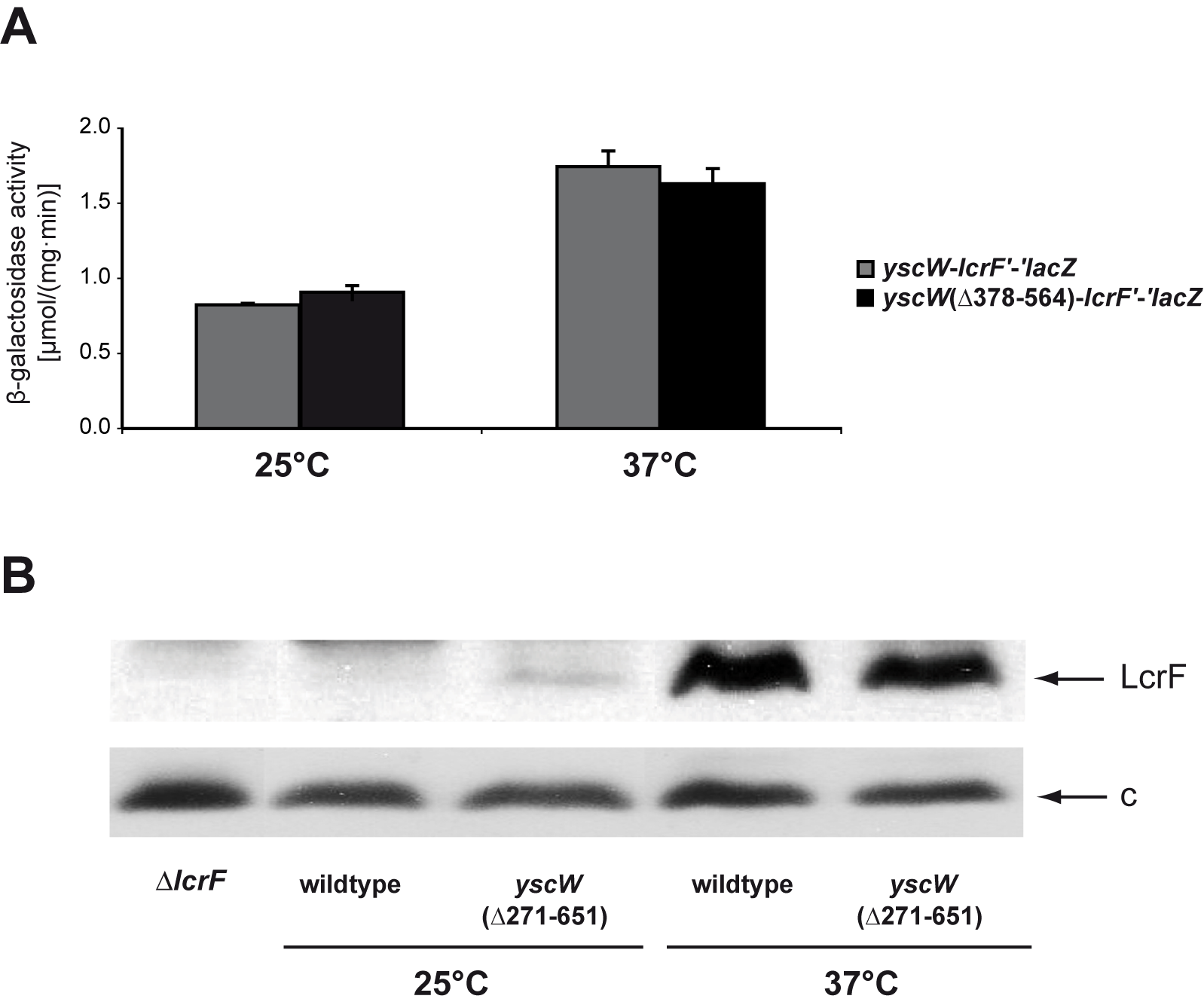

Supplement: Figure S4 — Influence of yscW on LcrF production. (A) YPIII harboring the different yscW-lcrF-lacZ or the yscW(Δ378–564)-lcrF-lacZ reporter plasmids pSF4 and pKB12 were grown overnight in LB medium at 25°C or 37°C. β-Galactosidase activity from overnight cultures was determined and is given in µmol min−1 mg−1 for comparison. The data represent the average ± SD from at least three experiments each done in duplicate. (B) YPIII and YP96 harboring a yscW(Δ271–651) were grown overnight in LB medium at 25°C or 37°C. Whole-cell extracts from overnight cultures were prepared and analysed by Western blotting with a polyclonal antibody directed against LcrF. A molecular weight marker is loaded on the left. A higher molecular weight protein (c) that reacted with the polyclonal antiserum was used as loading control. (TIF) [file ppat.1002518.s004.tif]

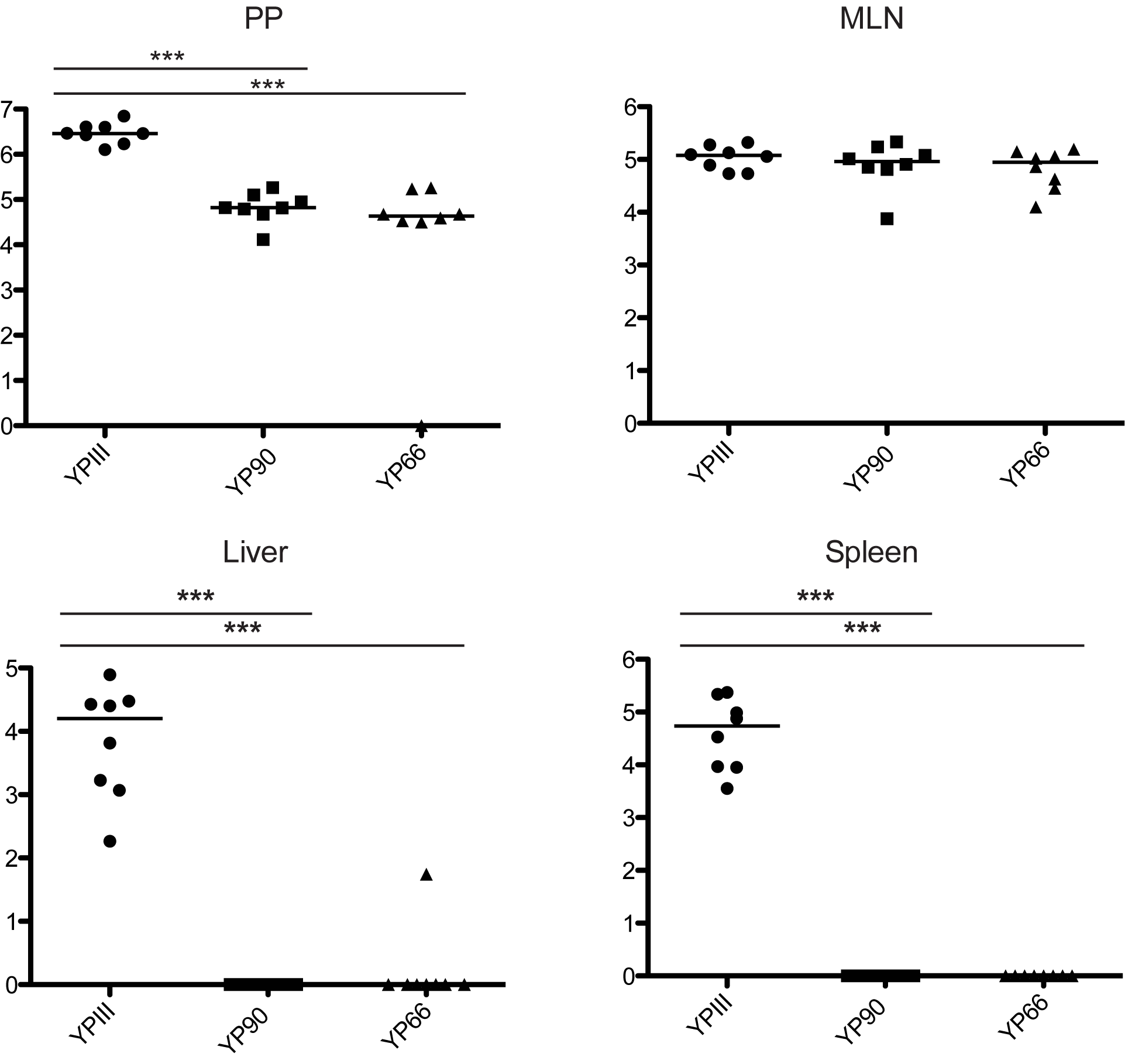

Supplement: Figure S5 — Influence of the lcrF RNA thermometer on tissue colonization by Y. pseudotuberculosis . Strains YPIII (wildtype), the ‘closed’ yscW-lcrF variant YP90, and YP66 (ΔlcrF) were infected intragastrically (5·108 CFU/mice) into BALB/c mice. After three days of infection, mice were sacrificed and the number of bacteria in homogenized host tissues and organs was determined by plating. Solid lines indicate the means. The statistical significances between the wildtype and the repressed and derepressed lcrF RNA thermometer variants were determined by the Student's t test. P-values: ***: <0.001. (TIF) [file ppat.1002518.s005.tif]

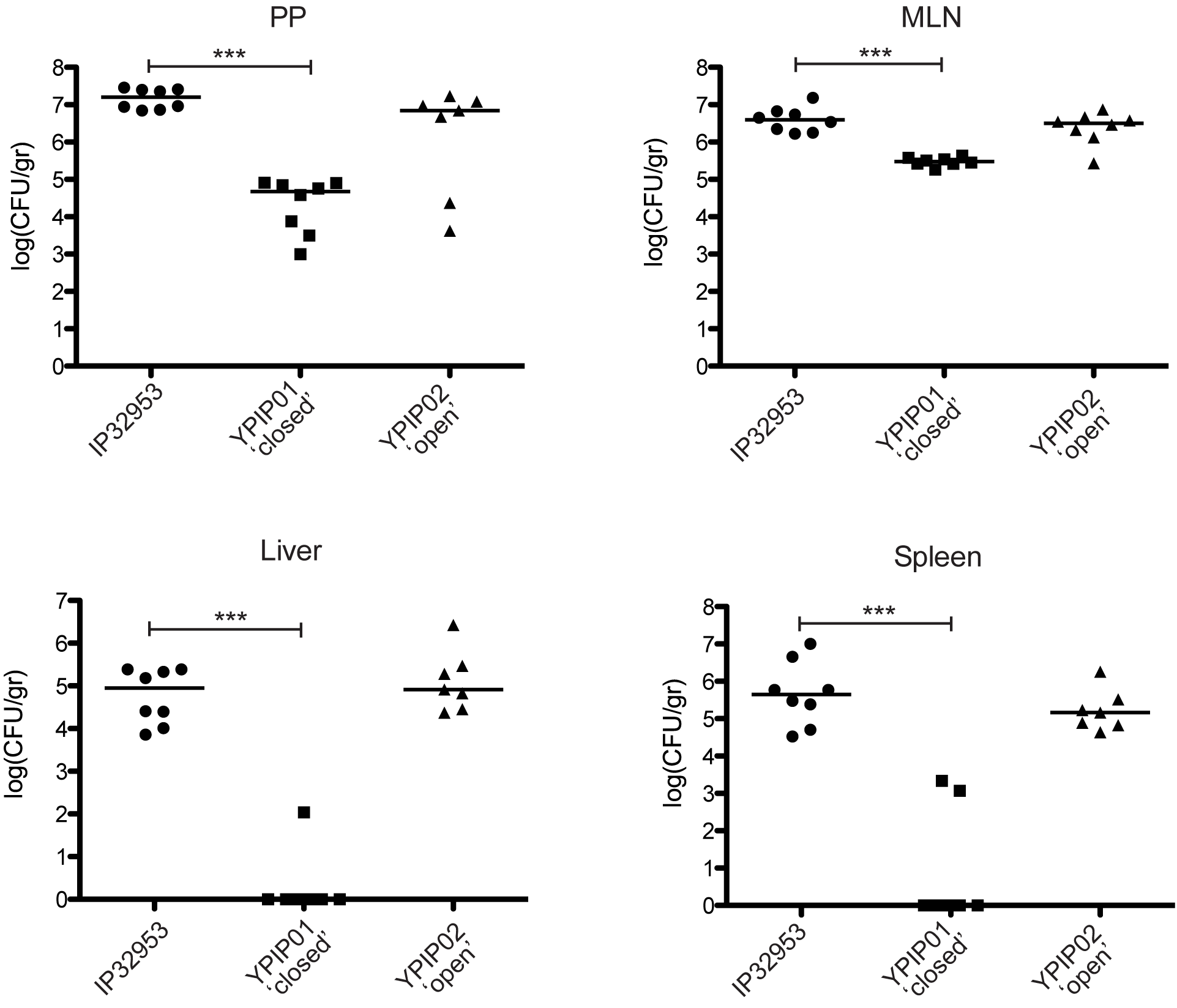

Supplement: Figure S6 — Influence of the lcrF RNA thermometer on tissue colonization by Y. pseudotuberculosis . Strains IP32953 (wildtype), the ‘closed’ and open yscW-lcrF variant YPIP01 and YPIP02 were infected intragastrically (5·108 CFU/mice) into BALB/c mice. After three days of infection, mice were sacrificed and the number of bacteria in homogenized host tissues and organs was determined by plating. Solid lines indicate the means. The statistical significances between the wildtype and the repressed and derepressed lcrF RNA thermometer variants were determined by the Student's t test. P-values: ***: <0.001. (TIF) [file ppat.1002518.s006.tif]
