# Supplementary material for: Concerted Actions of a Thermo-labile Regulator and a Unique Intergenic RNA Thermosensor Control Yersinia Virulence
Source: PLoS Pathog. 2012 Feb 16;8(2):e1002518. doi: 10.1371/journal.ppat.1002518 (PMC3280987; doi:10.1371/journal.ppat.1002518)
Supplement: Figure S7 — Multiple DNA sequence alignment of the yscW-lcrF operon of pathogenic Yersinia species. The nucleotides identical in the sequences are indicated with dots. The alignment was created using ClustalW. The coding sequence of yscW and lcrF are given in green, the intergenic region (RNA thermometer sequence) is given in blue. The promoter regions are bold and underlined, the transcriptional and translational start sites as well as the stop codons are indicated in bold. Y. pstb: Y. pseudotuberculosis; Y. pest: Y. pestis and Y. ent: Y. enterocolitica. (DOC) [file ppat.1002518.s007.doc]

*Y.pstb* YPIII 1 TAGGCTGCAATGTAACTAGGAATATGGTTAAAACAGATGACATTTTTGTTGGTCTATACT 60

*Y.pest* CO92 1 TAGGCTGCAATGTAACTAGGAATATGGTTAAAACAGATGACATTTTTGTTGGTCTATACT 60

*Y.ent* 8081 1 TAGGCTGCAATGTAACTAGGAATATGGTTAAAACAGATGGCATTTTTGTTGGTCTATACT 60

*Y.ent* O:3 1 TAGGCTGCAATGTAACTAGGAATATGGTTAAAACAGATGGCATTTTTGTTGGTCTATACT 60

*************************************** ********************

*Y.pstb* YPIII 61 ATTTTCTCTACTTTCTAGTCTATGTTTTT--ATCGGAAAAATGGGGTGATTAACACCGGC 118

*Y.pest* CO92 61 ATTTTCTCTACTTTCTAGTCTATGTTTTT--ATCGGAAAAATGGGGTGATTAACACCGGC 118

*Y.ent* 8081 61 ATTTCCTCTACTTTCTAGTCTATGTTTTTTTATCGGAAAAATGGGGTGATTAACACCGGC 120

*Y.ent* O:3 61 ATTTCCTCTACTTTCTAGTCTATGTTTTTT-ATCGGAAAAATGGGGTGATTAACACCGGC 119

**** ************************ *****************************

*Y.pstb* YPIII 119 TATTGGGAATATGTAATTTATTCGATATGGTTAACCAACAAGAGGTTCACTTATATTAGG 178

*Y.pest* CO92 119 TATTGGGAATATGTAATTTATTCGATATGGTTAACCAACAAGAGGTTCACTTATATTAGG 178

*Y.ent* 8081 121 TATTGGGAATATGTAATTTATTCGATATGGTTAACCAACAAGAGGTTCACTTATATTAGG 180

*Y.ent* O:3 120 TATTGGGAATATGTAATTTATTCGATATGGTTAACCAACAAGAGGTTCACTTATATTAGG 179

************************************************************

*Y.pstb* YPIII 179 AATATATGATGCGATTTAAAACCATATATACAGTATGGTAATTGTATTTCTCCTGTGCAT 238

*Y.pest* CO92 179 AATATATGATGCGATTTAAAACCATATATACAGTATGGTAATTGTATTTCTCCTGTGCAT 238

*Y.ent* 8081 181 AATATATGATGCGATTTAAA-CCATATATAAAGTATGGTGATTGTATTTCTCCTGTGCAT 239

*Y.ent* O:3 180 AATATATGATGCGATTTAAAACCATATATAAAGTATGGTGATTGTATTTCTCCTGTGCAT 239

******************** ********* ******** ********************

**-35**

*Y.pstb* YPIII 239 TTTACAATTAAACATAACTAGCACTAATTAGGATTAATCTC**TTGAC**TTTTTTTTGTTGAA 298

*Y.pest* CO92 239 TTTACAATTAAACATAACTAGCACTAATTAGGATTAATCTCTTGACTTTTTTTTGTTGAA 298

*Y.ent* 8081 240 TTTACAATTAAACATAACTAGCACTAATTAGGATTAATCTCTTGACTTTTTTT-GTTGAA 298

*Y.ent* O:3 240 TTTACAATTAAACATAACTAGCACTAATTAGTATTAATCTCTTGACTTTTTTT-GTTGAA 298

******************************* ********************* ******

**-10 +1**

*Y.pstb* YPIII 299 TACA**AATAAT**ACA**G**TATTGTCATTACTATTACATGTGTTTGTTTGCTGATTCCGATTCTA 358

*Y.pest* CO92 299 TACAAATAATACAGTATTGTCATTACTATTACATGTGTTTGTTTGCTGATTCCGATTCTA 358

*Y.ent* 8081 299 TACAAATAATACAGTATTGTCATTACTATTACATGTGTTTGTTTGCTGATTCCGATTCTA 358

*Y.ent* O:3 299 TACAAATAATACAGTATTGTCATTACTATTACATGTGTTTGTTTGCTGATTCCGATTCTA 358

************************************************************

*Y.pstb* YPIII 359 ACCAAACATCCTTTCTTT-ATGAAAGAAAGGATGTTTGGCTTTATATGCGCAAGGTGTGA 417

*Y.pest* CO92 359 ACCAAACATCCTTTTTTT-ATGAAAGAAAGGATGTTTGGCTTTATATGCGCAAGGTGTGA 417

*Y.ent* 8081 359 ACCAAACATCCTTTCTTTCGTGAAAGAAAGGATGTTTGGTTTTATACACACAAGGTGTGA 418

*Y.ent* O:3 359 ACCAAACATCCTTTCTTTCACGAAAGAAAGGATGTTTGGTTTTATACACGCAAGGTGTGA 418

************** *** ****************** ****** * **********

*Y.pstb* YPIII 418 TATTGCAGTAGCTAAATTAAATGAGTTCGTGGTGGACCCGTTGAGATAATTGGGAATGGG 477

*Y.pest* CO92 418 TATTGCAGTAGCTAAATTAAATGAGTTCGTGGTGGACCCGTTGAGATAATTGGGAATGGG 477

*Y.ent* 8081 419 TATTGCAGTAGCTAAATTAAATGGGTTCGCGGTGGACCCGTTGAGCTAATTGGGTATGGG 478

*Y.ent* O:3 419 TATTGCAGTAGCTAAATTAAATGAGTTCGCGGTGGACCCGTTGAGCTAATTGGGTATGGG 478

*********************** ***** *************** ******** *****

*Y.pstb* YPIII 478 TTGTTATTTTACAATAATAAATTTCACCACATATTGCGCGAACTCGGATTGCTATCATCT 537

*Y.pest* CO92 478 TTGTTATTTTACAATAATAAATTTCACCACATATTGCGCGAACTCGGATTGCTATCATCT 537

*Y.ent* 8081 479 TTGTTATTTTACAATAATAAATTTCACCACATATTGCGTGAACTCGGATTGCTATCATCT 538

*Y.ent* O:3 479 TTGTTATTTTACAATAATAAATTTCACCACATATTGCGTGAACTCGGATTGCTATCATCT 538

************************************** *********************

**start *yscW***

*Y.pstb* YPIII 538 AGCTATCTTCTTTGTGTGAACTCAAGGGAGGGACTGGC**GTG**AGTCGTATTATAGCACTCA 597

*Y.pest* CO92 538 AGCTATCTTCTTTGTGTGAACTCAAGGGAGGGACTGGCGTGAGTCGTATTATAGCACTCA 597

*Y.ent* 8081 539 GGCTATCTTCTTTGTGTGAATTCAAGGG-GAGACTGGCATGAGTCGTATTATAGCACTTA 597

*Y.ent* O:3 539 GGCTATCTTCTTTGTATGAATTCAAGGG-GAGACTGGCATGAGTCGTATTATAGCACTTA 597

************** **** ******* * ******* ******************* *

*Y.pstb* YPIII 598 TCATTTCTTTTCTATTAGTGGGGTGCGCTACCCCCCCAATGCCAGCTCAGCGTATTGTGG 657

*Y.pest* CO92 598 TCATTTCTTTTCTATTAGTGGGGTGCGCTACCCCCCCAATGCCAGCTCAGCGTATTGTGG 657

*Y.ent* 8081 598 TCATTTCTTTTCTATTAGTGGGGTGCGCTACCCCCCCAATGCCAGCTCAGCGTATTGTGG 657

*Y.ent* O:3 598 TCATTTCTTTTCTATTAGTGGGGTGCGCTACCCCCCCAATGCCAGCTCAGCGTATTGTGG 657

************************************************************

*Y.pstb* YPIII 658 GGGAGGTGCGTATGTCACGACCATTATCTCGCATAGCACACATTGATGTTAGTATGTTTG 717

*Y.pest* CO92 658 GGGAGGTGCGTATGTCACGACCATTATCTCGCATAGCACACATTGATGTTAGTATGTTTG 717

*Y.ent* 8081 658 GGGAGGTGCGTATGTCACGACCATTATCTCGCACAGCACACATTGATGTTAGTATATTTG 717

*Y.ent* O:3 658 GGGAGGTGCGTATGTCACGACCATTATCTCGCACAGCACACATTGATGTTAGTATATTTG 717

********************************* ********************* ****

*Y.pstb* YPIII 718 GGTTGTATGAGGGGAAAGTTCGAGAGGTTCAGCGCACTCATTTCGAAACAGGTAACCTAC 777

*Y.pest* CO92 718 GGTTGTATGAGGGGAAAGTTCGAGAGGTTCAGCGCACTCATTTCGAAACAGGTAACCTAC 777

*Y.ent* 8081 718 GGTTGTATGAGGGGAAAGTTCGAGAGGTTCAGCGCACTCGTTTCGAAACAGGTAACCTAC 777

*Y.ent* O:3 718 GGTTGTATGAGGGGAAAGTTCGAGAGGTTCAGCGCACTCGTTTCGAAACAGGTAACCTAC 777

*************************************** ********************

*Y.pstb* YPIII 778 CTTTATTCTTTTCTATAAAACTGAATCCAGCTCAACGCGGGGAAGGTGAACTTTACCTAC 837

*Y.pest* CO92 778 CTTTATTCTTTTCTATAAAACTGAATCCAGCTCAACGCGGGGAAGGTGAACTTTACCTAC 837

*Y.ent* 8081 778 CTTTATTCTTTTCTATAAAACTGAATCCAGCTCAGCGCGGGGAAGGTGAACTTTACCTAC 837

*Y.ent* O:3 778 CTTTATTCTTTTCTATAAAACTGAATCCAGCTCAACGCGGGGAAGGTGAACTTTACCTAC 837

********************************** *************************

*Y.pstb* YPIII 838 GGTCAACCCTCTCTTTTCCAGAGCGAGGAGTTCAGGCGGTGGCTCAGCAAAAGCTTACTG 897

*Y.pest* CO92 838 GGTCAACCCTCTCTTTTCCAGAGCGAGGAGTTCAGGCGGTGGCTCAGCAAAAGCTTACTG 897

*Y.ent* 8081 838 GGTCAACCCTCTCTTTTCCAGAGCGAGGGGTTCAGGCGGTGGCTCAGCAAAAACTTATTG 897

*Y.ent* O:3 838 GGTCAACCCTCTCTTTTCCAGAGCGAGGGGTTCAGGCGGTGGCTCAGCAAAAACTTATTG 897

**************************** *********************** **** **

*Y.pstb* YPIII 898 GTAAAAACAAAGTCGTTTTACAAATGATACCTAAAACATGTTATCCAAATTGCCAGTTAC 957

*Y.pest* CO92 898 GTAAAAACAAAGTCGTTTTACAAATGATACCTAAAACATGTTATCCAAATTGCCAGTTAC 957

*Y.ent* 8081 898 GTAAAAACAAAGTCGTTTTACAAATGATACCTAAAACATGTTATCCAAATTGCCAGTCAC 957

*Y.ent* O:3 898 GTAAAAACAAAGTCGTTTTACAAATGATACCTAAAACATGTTATCCAAATTGCCAGTCAC 957

********************************************************* **

**stop *yscW* intergenic region/ RNA thermometer**

*Y.pstb* YPIII 958 CTAATACCAGA**TAGGTGATTTATTATATTGGTTTTGGTTGCATTAATCGATGGTTGTACA** 1017

*Y.pest* CO92 958 CTAATACCAGATAGGTGATTTATTATATTGGTTTTGGTTGCATTAATCGATGGTTGTACA 1017

*Y.ent* 8081 958 CTAATACCAGATAGGTGATTTATTATATTGGTTTTGGTTGCATTAATCGATGGTTGTACA 1017

*Y.ent* O:3 958 CTAATACCAGATAGGTGATTTATTATATTGGTTTTGGTTGCATTAATCGATGGTTGTACA 1017

************************************************************

*Y.pstb* YPIII 1018 **TCGCACGCATAATAACTCAATACACCTCATTAGATAAATATATACAAGTTTTAGATTTTT** 1077

*Y.pest* CO92 1018 TCGCACGCATAATAACTCAATACACCTCATTAGATAAATATATACAAGTTTTAGATTTTT 1077

*Y.ent* 8081 1018 TCGCACGCATAATAACTCAATACACCTCATTAGATAAATATATACAAGTTTTAGATTTTT 1077

*Y.ent* O:3 1018 TCGCACGCATAATAACTCAATACACCTCATTAGATAAATATATACAAGTTTTAGATTTTT 1077

************************************************************

**start *lcrF***

*Y.pstb* YPIII 1078 **AGGACAGTATAACATTTATG**GCATCACTAGAGATTATTAAATTAGAATGGGTCACACCTA 1137

*Y.pest* CO92 1078 AGGACAGTATAACATTTATGGCATCACTAGAGATTATTAAATTAGAATGGGTCACACCTA 1137

*Y.ent* 8081 1078 AGGACAGTATAACATTTATGGCATCACTAGAGATTATTAAATTAGAATGGGCCACACCTA 1137

*Y.ent* O:3 1078 AGGACAGTATAACATTTATGGCATCACTAGAGATTATTAAATTAGAATGGGCCACACCTA 1137

*************************************************** ********

*Y.pstb* YPIII 1038 TATTTAAGGTTGTTGAGCATTCACAAGATGGCCTATATATTCTTTTGCAAGGTCAGATTT 1197

*Y.pest* CO92 1038 TATTTAAGGTTGTTGAGCATTCACAAGATGGCCTATATATTCTTTTGCAAGGTCAGATTT 1197

*Y.ent* 8081 1038 TATTTAAGGTTGTTGAGCATTCACAAGATGGCCTATATATTCTTTTGCAAGGTCAGGTTT 1197

*Y.ent* O:3 1038 TATTTAAGGTTGTTGAGCATTCACAAGATGGCCTATATATTCTTTTGCAAGGTCAGATTT 1197

******************************************************** ***

*Y.pstb* YPIII 1098 CATGGCAGAGCAGCGGTCAGACATATGATTTAGATGAGGGGAATATGCTGTTTTTGCGTC 1257

*Y.pest* CO92 1098 CATGGCAGAGCAGCGGTCAGACATATGATTTAGATGAGGGGAATATGCTGTTTTTGCGTC 1257

*Y.ent* 8081 1098 CATGGCAGAACAGCAGTCAGACATATGATTTAGATGAGGGGAATATGCTGTTTTTGCGTC 1257

*Y.ent* O:3 1098 CATGGCAGAACAGCAGTCAGACATATGATTTAGATGAGGGGAATATGCTGTTTTTGCGTC 1257

********* **** *********************************************

*Y.pstb* YPIII 1258 GTGGCAGCTATGCTGTTCGATGTGGTACAAAAGAACCCTGCCAATTACTTTGGATTCCAT 1317

*Y.pest* CO92 1258 GTGGCAGCTATGCTGTTCGATGTGGTACAAAAGAACCCTGCCAATTACTTTGGATTCCAT 1317

*Y.ent* 8081 1258 GTGGCAGCTATGCTGTTCGATGTGGTACAAAAGAACCCTGCCAATTACTTTGGATTCCAT 1317

*Y.ent* O:3 1258 GTGGCAGCTATGCTGTTCGATGTGGTACAAAAGAACCCTGCCAATTACTTTGGATTCCAT 1317

************************************************************

*Y.pstb* YPIII 1318 TACCCGGCAGTTTTTTGAGTACTTTTTTGCATCGCTTTGGTTCTTTGCTTAGTGAAATTG 1377

*Y.pest* CO92 1318 TACCCGGCAGTTTTTTGAGTACTTTTTTGCATCGCTTTGGTTCTTTGCTTAGTGAAATTG 1377

*Y.ent* 8081 1318 TACCAGGCAGTTTTTTGAGTACTTTTTTACATCGGTTTGGTTCTTTGCTTAGTGAAATTA 1377

*Y.ent* O:3 1318 TACCAGGCAGTTTTTTGAGTACTTTTTTACATCGGTTTGGTTCTTTGCTTAGTGAAATTA 1377

**** *********************** ***** ************************

*Y.pstb* YPIII 1378 GACGAGACAACTCCACACCCAAACCATTGTTAATTTTTAATATTTCACCAATATTATCAC 1437

*Y.pest* CO92 1378 GACGAGACAACTCCACACCCAAACCATTGTTAATTTTTAATATTTCACCAATATTATCAC 1437

*Y.ent* 8081 1378 GACGAGACAATTCCACACCTAAGCCATTGTTAATTTTTAATATTTCACCAATATTATCAC 1437

*Y.ent* O:3 1378 GACGAGACAATGCCACACCCAAGCCATTGTTAATTTTTAATATTTCACCAATATTATCAC 1437

********** ******* ** *************************************

*Y.pstb* YPIII 1438 AATCCATTCAAAATCTATGTGCCATATTGGAACGGAGTGATTTTCCGTCAGTATTAACGC 1497

*Y.pest* CO92 1438 AATCCATTCAAAATCTATGTGCCATATTGGAACGGAGTGATTTTCCGTCAGTATTAACGC 1497

*Y.ent* 8081 1438 AATCCATTCAAAATCTATGTGCCATATTGGAACGGAGTGATTTTCCGTCAGTATTAACGC 1497

*Y.ent* O:3 1438 AATCCATTCAAAATCTATGTGCCATATTGGAACGGAGTGATTTTCCGTCAGTATTAACGC 1497

************************************************************

*Y.pstb* YPIII 1498 AACTGCGTATTGAGGAATTACTGCTTTTGCTTGCCTTTAGCTCGCAAGGGACTTTATTTC 1557

*Y.pest* CO92 1498 AACTGCGTATTGAGGAATTACTGCTTTTGCTTGCCTTTAGCTCGCAAGGGACTTTATTTC 1557

*Y.ent* 8081 1498 AACTGCGTATTGAGGAATTACTGCTTTTGCTTGCCTTTAGCTCGCAAGGGACTTTATTCC 1557

*Y.ent* O:3 1498 AACTGCGTATTGAGGAATTACTGCTTTTGCTTGCCTTTAGCTCGCAAGGGACTTTATTCC 1557

********************************************************** *

*Y.pstb* YPIII 1558 TCTCGGCTCTGCGCCATTTAGGCAACCGCCCAGAAGAACGGTTGCAAAAATTTATGGAGG 1617

*Y.pest* CO92 1558 TCTCGGCTCTGCGCCATTTAGGCAACCGCCCAGAAGAACGGTTGCAAAAATTTATGGAGG 1617

*Y.ent* 8081 1558 TCTCGGCTCTACGCCATTTAGGCAACCGCCCAGAAGAACGGTTGCAAAAATTTATGGAGG 1617

*Y.ent* O:3 1558 TCTCGGCTCTGCGCCATTTAGGCAACCGCCCAGAAGAACGGTTGCAGAAATTTATGGAGG 1617

********** *********************************** *************

*Y.pstb* YPIII 1618 AAAATTATCTACAAGGGTGGAAGCTAAGCAAATTTGCGCGAGAATTCGGCATGGGATTAA 1677

*Y.pest* CO92 1618 AAAATTATCTACAAGGGTGGAAGCTAAGCAAATTTGCGCGAGAATTCGGCATGGGATTAA 1677

*Y.ent* 8081 1618 AAAATTATCTACAAGGGTGGAAACTAAGCAAATTTGCGCGAGAATTCGGCATGGGACTAA 1677

*Y.ent* O:3 1618 AAAATTATCTACAAGGGTGGAAACTAAGCAAATTTGCGCGAGAATTCGGCATGGGATTAA 1677

********************** ********************************* ***

*Y.pstb* YPIII 1678 CCACATTCAAAGAACTGTTTGGTACAGTTTATGGCATTTCACCACGCGCCTGGATAAGCG 1737

*Y.pest* CO92 1678 CCACATTCAAAGAACTGTTTGGTACAGTTTATGGCATTTCACCACGCGCCTGGATAAGCG 1737

*Y.ent* 8081 1678 CCACATTCAAAGAACTGTTTGGTACAGTTTATGGCATTTCACCACGCGCCTGGATAAGCG 1737

*Y.ent* O:3 1678 CCACATTCAAAGAACTGTTTGGTACAGTTTATGGCATTTCACCACGCGCCTGGATAAGCG 1737

************************************************************

*Y.pstb* YPIII 1738 AGCGACGTATTCTCTATGCTCACCAATTACTTCTTA-ATGGTAAGATGAGTATTGTTGAT 1796

*Y.pest* CO92 1738 AGCGACGTATTCTCTATGCTCACCAATTACTTCTTA-ATGGTAAGATGAGTATTGTTGAT 1796

*Y.ent* 8081 1738 AGCGACGTATTCTCTATGCTCACCAATTACTTCTTATATTGTAAGATGAGTATTGTTGAT 1797

*Y.ent* O:3 1738 AGCGACGTATTCTCTATGCTCACCAATTACTTCTTA-ATGGTAAGATGAGTATTGTTGAT 1796

************************************ ** ********************

*Y.pstb* YPIII 1797 ATTGCCATGGAAGCGGGGTTCTCGAGTCAGTCTTATTTCACTCAAAGTTATCGACGTCGC 1856

*Y.pest* CO92 1797 ATTGCCATGGAAGCGGGGTTCTCGAGTCAGTCTTATTTCACTCAAAGTTATCGACGTCGC 1856

*Y.ent* 8081 1798 ATTGCCATGGAAGCAGGGTTCTCGAGTCAGTCTTATTTCACTCAAAGTTATCGACGTCGC 1857

*Y.ent* O:3 1797 ATTGCCATGGAAGCAGGGTTCTCGAGTCAGTCTTATTTCACTCAAAGTTATCGACGTCGC 1856

************** *********************************************

*Y.pstb* YPIII 1857 TTCGGATGCACTCCAAGCCAAGCCCGTCTTACTAAAATAGCAACCACAGGCTAA 1910

*Y.pest* CO92 1857 TTCGGATGCACTCCAAGCCAAGCCCGTCTTACTAAAATAGCAACCACAGGCTAA 1910

*Y.ent* 8081 1858 TTCGGATGCACTCCCAGCCAAGCCCGTCTTACTAAAATAGCAACCACAGGCTAA 1911

*Y.ent* O:3 1857 TTCGGATGCACTCCCAGCCAAGCCCGTCTTACTAAAATAGCAACCACAGGCTAA 1910

************** ***************************************
