# Supplementary material for: Concerted Actions of a Thermo-labile Regulator and a Unique Intergenic RNA Thermosensor Control Yersinia Virulence
Source: PLoS Pathog. 2012 Feb 16;8(2):e1002518. doi: 10.1371/journal.ppat.1002518 (PMC3280987; doi:10.1371/journal.ppat.1002518)
Supplement: Table S1 — Oligonucleotides used in this study. The corresponding restriction sites are underlined, the T7 promoter region is given in italic, and introduced basepair substitutions are indicated in bold, the deletion of nucleotides is indicated by a short line, the sequence of kan resistance cassette is given in bold and underlined. (DOC) [file ppat.1002518.s008.doc]

**Table S1.** Oligonucleotides used in this study.

| **Number** | Sequence | **Site^a^** |
| --- | --- | --- |
| 1 | GCGGCGGTCGACCTCTTGGCGACAGCCATC | *Sal*I |
| 2 | GGGCGCGGATCCGCTAAGCAGACTATTTCAC | *Bam*HI |
| 3 | *GAAATTAATACGACTCACTATAGGG*GTGATTTATTATATTGGTTTTG |  |
| 4 | TGGCCAGTGATGCCATAAATGTTATAC | *Mls*I |
| 5 | TATACAAGTCCTAGATTTTTAGGACA |  |
| 6 | TGTCCTAAAAATCTAGGACTTGTATA |  |
| 7 | TATACAAAAATTAGATTTTTAGGACA |  |
| 8 | TGTCCTAAAAATCTAATTTTTGTATA |  |
| 9 | *GAAATTAATACGACTCACTATAGGG*GTGATTTATTATATTGGTTTTG |  |
| 10 | TGGCCAATGCTCAACAACCTTAAATATAGG | *Mls*I |
| 11 | TATACAAGTCCTAGATTTTTAGGACA |  |
| 12 | TGTCCTAAAAATCTAGGACTTGTATA |  |
| 13 | TATACAAAAATTAGATTTTTAGGACA |  |
| 14 | TGTCCTAAAAATCTAATTTTTGTATA |  |
| 15 | GGCGCTAGCAGCCGTAAGCATATATAAGCATGG | *Nhe*I |
| 16 | CCGGAATTCCTTGGACATTACATACTCC | *Eco*RI |
| 17 | TTTGCTAGCGTGATTTATTATATTGGTTTTG | *Nhe*I |
| 18 | TTTGAATTCGCCATCTTGTGAATGCTCAAC | *Eco*RI |
| 19 | CACCTCATTCCATAAATATATACAAGTTTTAG |  |
| 20 | CTAAAACTTGTATATATTTATGGAATGAGGTG |  |
| 21 | CCTCATTAGATAAATATATACAAAAATTAGATTTTTAGG |  |
| 22 | CCTAAAAATCTAATTTTTGTATATATTTATCTAATGAGG |  |
| 23 | CCTCATTAGATAAATATATACAAGTCCTAGATTTTTAGG |  |
| 24 | CCTAAAAATCTAGGACTTGTATATATTTATCTAATGAGG |  |
| 25 | CGCACGCATAATAACTGGGGACACCTCATTAG |  |
| 26 | CTAATGAGGTGTCCCCAGTTATTATGCGTGCG |  |
| 27 | CATTAGATAAATATCCCCAAGTTTTAGATTTTTAGG |  |
| 28 | CCTAAAAATCTAAAACTTGGGGATATTTATCTAATG |  |
| 29 | GGGGCGGCTAGCGTCGTATTATAGCACTCATC | *Nhe*I |
| 30 | TTTGAATTCGCCATCTTGTGAATGCTCAAC | *Eco*RI |
| 31 | GGGGCGGCTAGCGGTGAACTTTACCTACGGTCAACC | *Nhe*I |
| 32 | GCGGCCTGCAGGGCTGCAATGTAACTAGGAATATGG | *Pst*I |
| 33 | GCGGCCTGCAGGCTATAATACGACTCACGC | *Pst*I |
| 34 | GTTATACTGTCCTAAAAATCTAATGAGGTGTATTGAG |  |
| 35 | CTCAATACACCTCATTAGATTTTTAGGACAGTATAAC |  |
| 36 | GCGGCCTGCAGGGCTGCAATGTAACTAGGAATATGG | *Pst*I |
| 37 | GCGGCTGCAGCCATCTTGTGAATGCTCAACAACC | *Pst*I |
| 38 | GCGGCCTGCAGGGGGTGATTAACACCGGC | *Pst*I |
| 39 | GCGGCCTGCAGCAGTATGGTAATTGTATTTCTCC | *Pst*I |
| 40 | GCGGCCTGCAGCAGTATTGTCATTACTATTACATG | *Pst*I |
| 41 | GCGGCCTGCAGGCGCAAGGTGTGATATTGC | *Pst*I |
| 42 | GGTGATTTATTATACACCTCATTAGATAAATATATAC |  |
| 43 | CTAATGAGGTGTATAATAAATCACCTATCTGG |  |
| 44 | GCACTAGTGAAACAGGTAACCTACC | *Spe*I |
| 45 | GCGCATGCCTAAAGCAAAGAACCAAAGC | *Sph*I |
| 46 | GCACTAGTGCACTCATTTCGAAACAGG | *Spe*I |
| 47 | GCGCATGCCAAAAAAGTACTCAAAAAACTG | *Sph*I |
| 48 | GCACTAGTGAAAGTTCGAGAGGTTCAG | *Spe*I |
| 49 | GCGCATGCGTACTCAAAAAACTGCCGG | *Sph*I |
| 50 | CTCAATACACCTCATT**CC**ATAAATATATACAAGTTTTAGATTTTTAG |  |
| 51 | GTATATATTTAT**GG**AATGAGGTGTATTGAGTTATTATG |  |
| 52 | CATTAGATAAATATATAACAA**AAA**TTAGATTTTTAGGACAGTATAAC |  |
| 53 | CCTAAAAATCTAA**TTT**TTGTATATATTTATCTAATGAGG |  |
| 54 | GATAAATATATACAAGT**CC**TAGATTTTTAGGACAGTATAAC |  |
| 55 | CCTAAAAATCTA**GG**ACTTGTATATATTTATCTAATGAG |  |
| 56 | CATTAGATAAATAT**CCC**CAGTTTTAGATTTTAGGAC |  |
| 57 | CCTAAAAATCTAAAACTTG**GGG**ATATTTATCTAATGAGGTGTATTG |  |
| 58 | GCGCGCTGCAGGACTGGCGTGAGTCGTATTATAGC | *Pst*I |
| 59 | GCGCGCTGCAGGATTTTTAGGACAGTATAAC |  |
| 60 | GCGGCGTCTAGACCATTGAATCTTCACAATCTAATCCCG |  |
| 61 | TGGCCAATGCTCAACAACCTTAAATATAGG | *Mls*I |
| 62 | CCACGAACTCATTTAATTTAGC |  |
| 63 | CATTAGATAAGGTTTTAGATTTTAGATTTTTAG |  |
| 64 | CTAAAAATCTAAAACTTATCTAATG |  |
| 65 | GGGCGCGGTACCCAATATATTAAGGAACTTAAAATGAG | *Kpn*I |
| 66 | CGGCGCGGTACCGTATTATTTGTATTCAACAAAAAAAAG | *Kpn*I |
| 67 | CGGCGCGGTACCGAGTTCACACAAAGAAGATAG | *Kpn*I |
| 68 | GGGCGCGGTACCCGACTCACGCCAGTCCCTCCC |  |
| 69 | GGGCGCGGTACCCAGTATTGTCATTACTATTACATG |  |
| 70 | CCGGGCGACCCTGTTGATCG |  |
| 71 | GTCCTGGCCTGAATCGACAGCG |  |
| 72 | GGCTACGAAATGAGCATCGC |  |
| 73 | CTTTTGCTGAGCCAC-GCGAGATAATGGTCGTGACATACG |  |
| 74 | CGTATGTCACGACCATTATCTCGC-GTGGCTCAGCAAAAG |  |
| 75 | GCGGCGACTAGTGCACTAATTAGGATTAATCTCTTGAC | *Spe*I |
| 76 | CCAATATAATAAATCACCTATCTGGT-ACTCACGCCAGTCCCTCCC | *Sph*I |
| 77 | GGGAGGGACTGGCGTGAGT-ACCAGATAGGTGATTTATTATATTGG |  |
| 78 | GGGAGGGACTGGCGTGAGT-ACCAGATAGGTGATTTATTATATTGG |  |
| 79 | GCGGCGGAATTCGACAAAAACTGACTACCTGATG | *Eco*RI |
| 80 | GGGCCGCTCGAGGACTATTTCACATGTTGCCATAC | *Xho*I |
| 81 | CTACGCGACGAAATACTTTTTTTGTTTTGGCGTTAAAAGGTTTTCTTTATT**GTGTAGGCTGGAGCTGCTTC** |  |
| 82 | CGAGCTTGAGAAGCGACGCCGGACGCGCCCTAGCAGCGACATCCGGCCTCAG**CATATGAATATCCTCCTTAGT** |  |
| 83 | GCTCTATTATTACCTCAACAAACCACCCCAATATAAGTTTGAGATTACTAC**GTGTAGGCTGGAGCTGCTTC** |  |
| 84 | CAATAAAAAATCCCGCCGCTGGCGGGATTTTAAGCAAGTGCAATCTAC  AAAAG**CATATGAATATCCTCCTTAGT** |  |
| 85 | CCTGCGAGTTTATCTTGTTAGAATTATTACAACCATAGGTAGAAG**GTGTAG**  **GCTGGAGCTGCTTC** |  |
| 86 | GGGTATGTCTTCATGGCGAAAAAGTATAAAATTCTTAATAAACAGCCGGTT  ATAGCTCCGAAAGCG**CATATGAATATCCTCCTTAGT** |  |
| 87 | GCGGCCTGCAGGGCTGCAATGTAACTAGGAATATGG | *Pst*I |
| 88 | GCGGCTGCAGCCATCTTGTGAATGCTCAACAACC | *Pst*I |
| 89 | *ACTAAGGAGGATATTCATATG* CTGCTTAGCGCTGGTTAAG |  |
| 90 | GCGGCGGAGCTC CCATTCTGAACGAATGGTTTG |  |
| 91 | GCGGCCTGCAGCAATGAATTATCTCCTTAACG | *Pst*I |
| 92 | GCGGCGGCTGCAGTCTTTAGTCATTGTAAGCAC | *Pst*I |
