# Supplementary material for: Concerted Actions of a Thermo-labile Regulator and a Unique Intergenic RNA Thermosensor Control Yersinia Virulence
Source: PLoS Pathog. 2012 Feb 16;8(2):e1002518. doi: 10.1371/journal.ppat.1002518 (PMC3280987; doi:10.1371/journal.ppat.1002518)
Supplement: Table S2 — Primers used for the generation of deletion mutants. a The Y. pseudotuberculosis mutants were constructed by adding a kanamycin resistance cassette (Kan). Underlined bases correspond to the homologous nucleotides of the resistance gene. Rev: reverse primer; for: forward primer. (DOC) [file ppat.1002518.s009.doc]

**Table S2.** Primers used for the generation of deletion mutants.

| Mutant name^a^ | Primer name | Primer sequence |
| --- | --- | --- |
| YP50 | *ymoA*::Kan^a^ for | GGTTAATTGGTTGTAACACTGGCTGCTTAGCGCTGGTTAAGACACACAACGTTGAGCCGATAATCTCT ATCG |
|  | *ymoA*::Kan^a^ rev | GCAAAGCAAAAGTTCAAAATCACCGGTTTTTCTTCTCGATATACAAATTAATATTGGTGGAACTAT CCC |
|  | *ymoA* for | CGATAGACAGCTGTATTTATATG |
|  | *ymoA* rev | CCTGTATTATCACTTTCCTGC |
| YP66 | *lcrF*::Amp for | CCTCATTAGATAAATATATACAAGTTTTAGATTTTTAGGACAGTATAACATTTGGGCCTCGTGATACGCC |
|  | *lcrF*::Amp rev | GGTTGCTATTTTAGTAAGACGGGCTTGGCTTGGAGTGCATCCGAAGCGAGTAAACTTGGTCTGACAG |
| KB1 | *stpA*::kan for | CTACGCGACGAAATACTTTTTTTGTTTTGGCGTTAAAAGGTTTTCTTTATTGTGTAGGCTGGAGCTGCTTC |
|  | *stpA*::kan rev | CGAGCTTGAGAAGCGACGCCGGACGCGCCCTAGCAGCGACATCCGGCCTCAGCATATGAATATCCTCCTTAGT |
| KB3 | *hns*::kan for | GCTCTATTATTACCTCAACAAACCACCCCAATATAAGTTTGAGATTACTACGTGTAGGCTGGAGCTGCTTC |
|  | *hns*::kan rev | CAATAAAAAATCCCGCCGCTGGCGGGATTTTAAGCAAGTGCAATCTAC  AAAAGCATATGAATATCCTCCTTAGT |
| KB4 | *hha*::kan for | CCTGCGAGTTTATCTTGTTAGAATTATTACAACCATAGGTAGAAGGTGTAG  GCTGGAGCTGCTTC |
|  | *hha*::kan rev | GGGTATGTCTTCATGGCGAAAAAGTATAAAATTCTTAATAAACAGCCGGTT  ATAGCTCCGAAAGCGCATATGAATATCCTCCTTAGT |
| kan*_ymoA_* | *kan_1_* for | GGTGATTTTGAACTTTTGCTTTG |
|  | *kan_1_* rev | CCAGTGTTACAACCAATTAACC |
| Kan*_pKD4_* | *kan_2_* for | GTGTAGGCTGGAGCTGCTTC |
|  | *kan_2_* rev | CATATGAATATCCTCCTTAGTTCC |
